# A Review of Mathematical and Computational Methods in Cancer Dynamics

**DOI:** 10.3389/fonc.2022.850731

**Published:** 2022-07-25

**Authors:** Abicumaran Uthamacumaran, Hector Zenil

**Affiliations:** ^1^ Department of Physics, Concordia University, Montreal, QC, Canada; ^2^ Machine Learning Group, Department of Chemical Engineering and Biotechnology, The University of Cambridge, Cambridge, United Kingdom; ^3^ The Alan Turing Institute, British Library, London, United Kingdom; ^4^ Oxford Immune Algorithmics, Reading, United Kingdom; ^5^ Algorithmic Dynamics Lab, Karolinska Institute, Stockholm, Sweden; ^6^ Algorithmic Nature Group, LABORES, Paris, France

**Keywords:** cancer, dynamical systems, complexity science, complex networks, information theory, inverse problems, algorithms, systems oncology

## Abstract

Cancers are complex adaptive diseases regulated by the nonlinear feedback systems between genetic instabilities, environmental signals, cellular protein flows, and gene regulatory networks. Understanding the cybernetics of cancer requires the integration of information dynamics across multidimensional spatiotemporal scales, including genetic, transcriptional, metabolic, proteomic, epigenetic, and multi-cellular networks. However, the time-series analysis of these complex networks remains vastly absent in cancer research. With longitudinal screening and time-series analysis of cellular dynamics, universally observed causal patterns pertaining to dynamical systems, may self-organize in the signaling or gene expression state-space of cancer triggering processes. A class of these patterns, strange attractors, may be mathematical biomarkers of cancer progression. The emergence of intracellular chaos and chaotic cell population dynamics remains a new paradigm in systems medicine. As such, chaotic and complex dynamics are discussed as mathematical hallmarks of cancer cell fate dynamics herein. Given the assumption that time-resolved single-cell datasets are made available, a survey of interdisciplinary tools and algorithms from complexity theory, are hereby reviewed to investigate critical phenomena and chaotic dynamics in cancer ecosystems. To conclude, the perspective cultivates an intuition for computational systems oncology in terms of nonlinear dynamics, information theory, inverse problems, and complexity. We highlight the limitations we see in the area of statistical machine learning but the opportunity at combining it with the symbolic computational power offered by the mathematical tools explored.

## Introduction

Cancer is the second leading cause of disease-related death globally, and a tremendous burden to progressive medicine. Deciphering the minimal set of interactions in the complex multiscale networks driving cancer gene expression and signaling remains an intractable problem due to its collective emergent behaviors, including phenotypic (epigenetic) plasticity, intra-tumoral heterogeneity, therapy resistance, and cancer stemness (1). Cancer is essentially a genetic disease and given the vast amounts of scientific works on the genetic and chromosomal instabilities/mutations driving tumorigenesis and cancer evolutionary dynamics, we shall not directly explore these conventional realms herein. Rather the review will focus on the cell fate dynamics emerging from these genetic/chromosomal instabilities, and hallmarks of cancer progression/adaptivity. Further, molecular heterogeneity is observed across many scales of cancer cybernetics, necessitating multiomics and multimodal profiling methods to dissect cancer ecosystems (2). In this regards, systems biology and computational medicine have paved many powerful tools in single-cell analyses including network theory, data science, statistical machine learning, and multivariate information theoretics. However, current approaches in network medicine rely on static (single time-point) visualizations of spatial cancer cell-signaling and gene expression profiles. These predominant snapshot approaches are fundamental limiting factors in the advancement of precision oncology since they are causal agnostic, i.e., they remove the notion of *time* (*dynamics*) from cancer datasets. Instead, pseudotemporal ordering techniques are used to infer gene expression patterns and cell fate trajectories in cancer processes on dimensionality-reduced pattern spaces. Although these static patterns may provide us insights into statistical correlations in complex cancer processes, statistical *correlation does not imply causation*. The lack of time-series measurements in single-cell multi-omics (e.g., gene expression dynamics, protein oscillations, histone marks spreading, etc.) and cell population fluctuations (i.e., ecological dynamics), in patient-derived tumor and liquid biopsies, remains a central roadblock in reconstructing cancer networks as complex *dynamical systems*.

Let us suppose we do have time-sequential measurements to infer signaling and gene expression dynamics in cancer networks by the methods suggested herein. What kinds of patterns emerge in time-series which cannot be inferred from the currently predominant snapshot approaches? How do we detect these causal patterns in cancer dynamics? The review was written precisely to address these questions and provide a general intuition for nonlinear dynamics, chaos, and complexity in cancer research. The central dogma in systems biology remains that gene expression dynamics are *stochastic processes*. However, unlike stochastic systems, *deterministic chaos*, although difficult to distinguish from randomness, has a defined causal pattern in state-space (3). Chaotic systems exhibit an underlying (multi)fractal topology, defined as strange attractor(s). Unlike randomness, these causal patterns allow the short-term predictions of the chaotic system’s time-evolution (up to some Lyapunov time) and their global state-space trajectory (orbit). Chaotic systems also exhibit patterns of *emergent behaviors*, i.e., collective patterns and structures which are unpredictable from the individual components (3).

The primary theme of the paper is, if chaotic behavior or complex dynamics plays an important role within cancer dynamics, how do we characterize chaos/complexity and distinguish it from randomness? The review comprises of a detailed discussion of tools from complex systems science and nonlinear dynamics (dynamical systems theory) to decode multiscale processes and behavioral patterns in cancer cellular dynamics. Various detecting tools, measures and algorithms exist which remain under-explored in (computational) systems medicine. Complex multiscale/multiomic networks driving cancer emergence/progression are discussed across different scales of cancer processes, from gene regulatory networks to epigenetic stemness/plasticity networks. An intuition for cellular oscillations (both intracellular flows and population dynamics) and causality inference in cancer cell fate dynamics are presented under the lens of complex systems. The remaining bulk of the paper is devoted to a step-by-step blueprint of algorithms and tools to capture multiscale chaotic dynamics (if they exist) within cancer cell signaling and cellular processes. Dynamical systems theory is a relatively new framework to most cancer researchers further stressing the lack of time-series cancer datasets. As such, some codes for a selected set detection tools/algorithms are provided in the **Appendix**. A summary table of some inference methods discussed are also provided as **Table 1** in the **Appendix**. Furthermore, traditionally, signaling refers to protein-protein interactions or signal transduction pathways in cell communication networks. Examples of such signaling includes physiological cybernetics such as psychoneuroendocrine control, immune-inflammatory pathways, neurotransmitter dynamics, extracellular vesicles-mediated communication networks, and other receptor-ligand regulatory feedback loops/signaling cascades. However, we may simply refer to various scales of cancer cybernetics, including cancer-immune population dynamics, protein density fluctuations, epigenetic patterns/chromatin modifications, metabolomics, and gene expression (transcriptional) dynamics, to name a few, simply as *signaling* herein in reference to signal (information) processing in control systems (cybernetics). Due to the limited space allocated to the comprehensive review, additional information such as biological insights into epigenetic complexity, some fine-details of the mathematical treatments/methods, and prospective techniques for the acquisition of time-resolved multicell data are provided In the the **Appendix**.

**Table 1:** GLOSSARY.

METHOD	DESCRIPTION
Takens’ theorem	A technique for embedding the time-series signal in state-space using a time-delay in one of its coordinates. Convergent Cross Mapping is an embedding algorithm implementing Takens’ theorem, applicable on complex networks. The technique has dimensionality limits and hence, should only be limited to a few signals with predicted chaotic dynamics.
Denoising Algorithms	Any algorithm intended for noise reduction. Can range from filtering and preprocessing tools (interpolation, smoothening, etc.) to wavelet-analysis methods. Imputation algorithms are emerging as popular candidates. Not discussed in detail since it consists of a wide range of algorithms, the applicability of which depends on the type of dataset and system of interest.
Lyapunov Exponents	Measures how fast two initially close points on a chaotic trajectory exponentially diverge apart in time. Positive Lyapunov exponent(s) are characteristic signatures of chaos.
Fractal Dimension	Fractals are the geometry of chaos. A fractal is a geometric pattern exhibiting statistical self-similarity (i.e., power law scaling) across many length and time scales with a fractional (non-integer) dimension. It is used as a measure of irregularity, roughness, and complexity. Some algorithms to estimate the Fractal Dimension include the Box-counting method, Fourier analysis-based approaches, and the sandbox method.
Multifractal Analysis	If more than one fractal dimension is required to describe the complexity of the system, multifractal analysis is required. These approaches are most applicable for time-series analysis. The local Holder exponents and the Hurst index are pertinent measures. Wavelet Transform-based methods remain the most popular tools for identifying these multifractal statistics.
Fast-Fourier Transform (FFT)	The frequency and power spectra of time-series signals can be acquired using FFT. The FFT algorithm decomposes a time-series into its constituent frequencies. Chaotic systems generally exhibit a broad frequency spectrum.
Criticality	Power laws are indicators of critical dynamics, a state of hierarchical self-organization poised between regularity and chaos. When certain complex systems surpass their critical point, they gravitate towards chaotic dynamics. The Ising model is discussed as a powerful tool to model criticality in cancer gene expression and patterns of network dynamics.
Entropy	Maximal entropy and a positive entropy rate are observed in dynamical systems exhibiting increased chaotic flows in phase-space. They could be indicators of phase-transitions to chaotic dynamics and/or the birth of complex attractors. However, entropy is not a robust measure of network (graph) complexity and may fail to distinguish randomness from chaoticity.
Computational Modelling and Simulations	The pairing of simulations/computational modelling with data science is the central principle of complexity science. Herein stochastic simulations such as the Monte Carlo methods and Gillespie algorithm were discussed for simulating chemical kinetics and molecular dynamics.
Recurrent Neural Networks (RNN)	Reservoir Computing (RC) networks and liquid neural networks are the state-of-the-art Deep Learning Networks for time-series forecasting and spatiotemporal prediction of chaotic dynamics from complex, multidimensional datasets.
Algorithmic Complexity	Also known as the Kolmogorov complexity [K(s)], is a measure of the length of the shortest description of a dataset (e.g., a string, an array, a network, or dynamical system) or the shortest program needed to generate the dataset. Various algorithms exist for estimating the K-complexity. CTM and BDM (Block Decomposition Method) are alternatives to statistical compression algorithms and are native to n-dimensional complexity.

## Complex Dynamics

*Complexity theory* is an interdisciplinary paradigm in systems science merging nonlinear dynamics, statistical mechanics, information theory, and computational physics. It deals with the study of *whole systems* which exhibit emergent behavioral patterns, often due to their multi-scale *nonlinear interactions*, and multi-nested feedback loops. Therefore, *complex systems* are (in general) nonlinear feedback systems (including computational systems) with many interacting parts which give rise to collective behaviors (i.e., emergence) (80). Complex systems or their signature, emergence, may be best defined by the non-reductionistic Aristotelian dictum *the whole is more than the sum of its parts* (7). Complexity theory is thus the quantitative study of collective processes, patterns, and behaviors in complex systems. *Chaotic systems* are at the heart of biological/physiological complex systems and warrant our deepest attention.

The universality of chaotic dynamics in physiological control systems has been well-established. Based on the findings of the Jacob-Monod model of lac operon regulation, Goodwin, a student of Waddington, first derived an oscillatory model of gene regulatory networks to describe negative feedback loop oscillators as seen in a wide range of biological processes including circadian rhythms, enzymatic processes, developmental biology, and cell cycle dynamics (81). Later, the Mackey-Glass equations, a set of first-order nonlinear delay-differential equations, demonstrated that complex dynamics ranging from limit-cycle oscillations to chaotic attractors can emerge in respiratory and hematopoietic diseases (82). The most prominent examples of chaotic dynamics are found within cardiac oscillations (83–85). The works of Winfree and Kuramoto further extended the study of biological oscillators in physiological control processes/rhythms (46, 86). Chaotic oscillations have also been well-studied in glycolytic oscillations and cellular calcium fluxes. For instance, tumor glycolytic oscillations have been experimentally suggested to confer adaptive cellular behaviors such as therapy resistance in tumor ecosystems. In this model system, Pomuceno-Ordunez et al. (87) investigated the effects of pulses and periodic glucose deprivation in a kinetic model of HeLa cells glycolysis. A system of ordinary differential equations were obtained from the model to quantify the glycolytic oscillations. Various measures were used to assess the complex dynamics including ability analysis of the steady state, stroboscopic analysis, and Lempel-Ziv index. The study concluded that periodic glucose pulses can lead to an increase in the energy charge, while periodic glucose deprivation of the tumor ecosystem prevented the increase in the complexity of glycolytic oscillations and caused a decrease in the cellular energy charge of tumor cells (87). However, it should be emphasized that complex tumor ecosystems such as GBM exhibit adaptive heterogeneity and phenotypic plasticity amidst a diverse range of transcriptional and metabolic cellular states. While some cellular states may be glycolytic phenotypes, others may favor oxidative phosphorylation, and others are inclined towards other metabolic programs.

Further, the detection of chaotic oscillations in other cellular rhythms such as the circadian clock remain experimentally dormant. Only mathematical models and numerical simulations have by far shown the emergence of chaotic behavior at the level of clock protein oscillations in simpler model systems like Drosophila (88). The detection of intracellular chaotic oscillations in proteins and genetic networks, is recently emerging as a *paradigm shift* in complexity science (18, 19). Most biological systems at varying length and time scales, including networks of genes, proteins, and populations of cells, behave like coupled nonlinear oscillators (16, 17). In principle, chaotic oscillations can arise in these biological oscillators (16). In the context of cancer networks, there are many timescales and interconnected regulatory feedback loops manifesting time-delays in their oscillatory dynamics. The time-delays may give birth to signaling cascades and a symphony of complex dynamics, including chaotic oscillations (15, 17). For instance, calcium oscillations within cells are in the timescales of seconds, whereas protein oscillations such as transcription factor oscillations, the cell cycle, and circadian rhythms span from hours to days. This is the key insight cancer researchers should be aware of, that oscillatory dynamics occur in all length and time scales, including networks of gene expression (transcriptional dynamics), protein signals, multicellular networks, and ecological/population dynamics (e.g., tumor-immune predator-prey systems). When these oscillations become aperiodic and irregular, they may either be stochastic (random) or chaotic. However, unlike randomness, chaotic flows have an underlying causal pattern, a structure, in state-space to which their irregular trajectories are confined to (15). The methods for detecting different behavioral regimes in the experimental time-traces of cancer signals depend on the timescales of the oscillations and the multi-nestedness (interactions) of the complex network patterns or dynamics they form. Thus, the resolution of the time-series datasets acquired must also be considered in chaos/complex dynamics discovery.

The time-series signal, whether it be the oscillation of a single protein, a protein concentration density during cellular patterning, or gene expression dynamics, of cancer cells, can be represented as a state-vector *X*(*t*). For instance, the gene expression matrix acquired from a single-cell RNA-Seq experiment is a state vector at a given time point t. The state-space, also known as phase-space, determines the set of all possible values of the signal’s state-vector (15). Any state of the dynamical system at a moment frozen in time can be represented as a point in phase space. All the information about its position and velocity is contained in the coordinates of that fixed-point. As the system evolves, the point would trace a trajectory in phase space (15). In the context of the given example, the state-space would describe the entire range of possibilities in the oscillator(s), or all the possible gene-gene network configurations described by the count matrix. The state-space reconstruction of the signals’ time-traces (trajectories) exhibit a set of universal patterns called *attractors*. Attractors are self-organized causal structures governing the fate of a dynamical system in state-space. They are finite regions bound to state-space to which the trajectories of the dynamical system are confined to or pulled towards (i.e., attracted to) (Note: the opposite flow analog also exists, repellors, regions in state space from which the system is pushed away from) (15). The detection of attractors provides a route to reduce the combinatorically vast state-space of all network configurations conferring cancer states towards a finite set of values. However, only fixed-point attractors (equilibrium points), the simplest of attractors, are analytically solvable.

In the chaotic and complex regimes of dynamical systems, multiple attractors may self-organize for a wide range of initial conditions. Some attractors may entangle with those nearby to form complex webs of attractors. We need three dimensions (or higher) to analyze chaotic attractors, and hence, observing a single protein oscillation in time as a one- dimensional system is insufficient to detect chaotic behavior (15). As such, the most effective classical method for chaotic behavior detection is to embed the time-trace signal onto state-space by Takens’ theorem and quantify its Lyapunov exponents and fractal dimension. *Time-delay embedding* allows us to reconstruct a higher dimensional space of the protein flows or gene expression. However, there may be still smearing by noise. Denoising algorithms can be used as a filtering and pre-processing step prior to the attractor embedding. Denoising algorithms can be of many sorts from basic normalization techniques to wavelet analysis and imputation techniques, used for noise reduction in the dataset. From the time-embedded signaling data, one can identify two points that are very close in the phase space and subsequently measure the initial, exponential separation away from each other (15). This procedure determines the spectrum of Lyapunov exponents, where the presence of one or more positive Lyapunov exponents imply chaotic behavior (15). The presence of a chaotic attractor is further confirmed by assessing the embedded attractor’s fractal dimension in state-space (15). However, when large complex datasets in the order of thousands of genes or proteins within thousands of cells are considered, as is the case for patient-derived tumor/liquid biopsies and their single-cell analyses, the application of these embedding techniques and traditional chaotic behavior measures may not be sufficient due to dimensionality constraints. Therefore, as will be discussed, machine learning algorithms and algorithmic information dynamics are suggested as robust tools for mapping complex dynamics and inferring chaotic behavior in larger multidimensional datasets pertinent to systems oncology.

Chaotic behavior implies long-term unpredictability, irregularity, and complexity, making the disease difficult to treat. A healthy cell phenotype may correspond to a stable attractor in state space such as a fixed-point or a limit cycle (oscillations). However, a cell if found to be a chaotic attractor, is an unstable state with irregular and aperiodic signaling dynamics. Chaotic dynamics in certain biological oscillations may be robust biomarkers or patterns for diseases. An emerging paradigm in the study of complex diseases, such as cancers, is that chaotic dynamics can emerge in biological oscillators such as gene and protein networks. According to mathematical and computational models, the emergence of chaotic attractors in complex cancer processes have been suggested as indicators of therapy resistance, cancer relapse, emergence of aggressive phenotypes, increased phenotypic plasticity, and metastatic invasion (19, 89, 90). However, most of these studies were limited to cell population dynamics. Further, Huang et al. (91) were amidst the first to suggest using transcriptomic analyses that cancer cell fates are aberrant, embryonic-stem cell like attractors of the Waddington developmental landscape (78). Further, they suggested that cancer stem cells occupy higher energy states of the landscape, thus representing more complex attractors with higher differentiation potency (91).

Chaotic dynamics at the level of protein and gene oscillations may also confer dynamical heterogeneity and adaptive survival in individual cell states to withstand extreme environmental conditions due to their large signaling fluctuations (19). As such, it is further suggested here *chaotic attractors may be signature hallmarks of cancer stemness.* Measuring chaotic attractors then provides a solution to forecast the complex adaptive behaviors and dynamics of the disease system. The chaotic attractor provides a control system framework to reprogram the disease state dynamics in signalling state-space. If chaotic behavior emerges or is at the origin in cancer signaling dynamics, two fundamental questions arise: (1) How do we detect chaotic behavior of or in cancer cells? More specifically, how do we detect strange attractors in the (multiomic) signaling state-space of cancer networks? and (2) How do we distinguish chaotic oscillations from stochastic oscillations (randomness) or noise in the signaling state-space? To address these questions, a brief survey of tools and methods to detect strange attractors in cancer signaling state-space are outlined in this paper. While chaotic oscillations may occur in any cancer-related process, we will primarily focus our attention to the complex networks steering cancer cell fate dynamics and differentiation processes.

## Oscillations and Cell Patterning Systems

Given the reconstruction of the complex network patterns/dynamics steering cancer cell fate decision-making, we must understand how their regulatory feedback loops behave in time (i.e., oscillations). As mentioned, cells, genes, and proteins, can be treated as physiological oscillators. Thus, a brief intuition for oscillations is required to understand the use of chaotic-behavior detection tools, in the context of forecasting cancer cell signaling/dynamics. Cancers are essentially characterized by their abnormal, uncontrolled cell division due to chromosomal/genomic instabilities. The tumor suppressor transcription factor p53 is highly conserved at the protein level and plays a key role in DNA damage response. It is a master regulator of the cell cycle and hence, cell proliferation. In cancer cells, the TP53 gene is mutated (loss of function) in about 50% of all cancers and serves as the critical bifurcation point for tumorigenesis on the cell developmental/differentiation landscape (92). The core p53-MDM2 negative feedback loop shows that the synthesis-degradation kinetics of p53 and MDM2 governs their oscillations within cells. The oscillations of this regulatory feedback mechanism are essential for the signaling dynamics of all other intertwined proteins and genes regulating cell homeostasis and cell cycle control. Within this circuit, p53 transcriptionally activates *mdm2*. Mdm2, in turn, negatively regulates p53 by both inhibiting its activity as a transcription factor and by enhancing its degradation rate. Models of negative feedback loops, such as between p53 and Mdm2, suggest that they can generate an *oscillatory behavior* due to a time delay between the two proteins activity (conformation states). However, with DNA damage (as in the case of tumors harboring~ hundreds of mutations), excessive continuous p53 oscillations are observed (92).

Fluorescently tagged fusion proteins can be used for the time-lapse imaging of these proteins and quantify their oscillatory dynamics within cells. For different parameters of the feedback loop, the dynamics can show either a monotonic response, damped oscillations, or undamped oscillations. The stronger the coupling interactions between the proteins, the more oscillatory the dynamics tend to be. The kinetic parameters act as damping or signal-amplifying coefficients. For instance, high basal degradation rates of the proteins act as damping coefficients of the oscillations. To illustrate an experimental approach to measuring cellular protein oscillations, Geva-Zatorsky et al. (93) experimentally investigated fluorescently labelled p53 and Mdm2 dynamics in living breast cancer (MCF7) cells with perturbation analysis by gamma irradiation. In a large fraction of cells, they found undamped oscillations of p53-CFP and Mdm2-YFP, which lasted for at least ∼3 days post- gamma irradiation. Aside from the noise fluctuations in cell-cell variability, two characteristic properties were found in the protein oscillations: (1) the oscillation amplitudes fluctuated widely, yet the oscillation frequency was much less variable, and (2) while some cells regularly oscillated, other cells showed a dynamic fluctuation of protein levels (i.e., signaling heterogeneity) indicating the presence of complex, irregular oscillations. Essentially, the negative feedback loop amplified slowly varying noise in the protein production rates at frequencies near the resonance frequency of the feedback loop. However, none of these studies performed chaotic behavior detection such as time-delay embedding of the signal followed by calculation of Lyapunov exponents nor computing the fractal dimension of observed attractors. The description of the fluctuations and increases in cellular oscillations were qualitative in most part.

Similarly, many embryonic developmental factors (i.e., morphogens) involved in cellular patterning systems are known to exhibit oscillatory dynamics. The oscillatory states of these signals show typical time periods of a few hours (1-4 hrs) and are referred to as ultradian oscillations (94). A key example of ultradian oscillations is the coupling of Wnt and Notch signaling (95). One of the theoretical frameworks for understanding these protein/gene oscillations in developmental pattern formation has been laid by the clock-and-gradient, or clock-and-wavefront model, originally proposed by Cooke and Zeeman (96). The dynamic signal encoding based on relative timing of oscillatory protein signals are essential for the development of the embryo. It has been shown that a phase-shift in morphogen oscillations fine-tune segmentation in the developing embryo (95). These morphogens are aberrantly expressed in cancer stem cells and are key signaling factors of the cancer stem cell niche (20; 97). However, the experimental study of morphogen oscillations *via* time-lapse imaging within pathological cell states (such as cancer stem cells) remain nearly absent. Their oscillatory dynamics have not been investigated experimentally in time-series cancer stem cell differentiation or during cancer cell fate decisions such as proliferation and differentiation dynamics. Moreover, the detection of quantitative cellular behavioral techniques as advocated herein are virtually unknown to most cancer researchers investigating protein-mediated pattern formation. Therefore, the lack of experimental time-series cancer datasets (due to technological limitations) and a lack of dynamical systems theory applications in cancer research go hand in hand and limit our understanding of how complex dynamics may be orchestrating tumor patterning systems.

As explained, when the amplitude of an oscillation by coupling to some external signal increases beyond a critical threshold, chaotic dynamics can emerge as indicated by aperiodicity/irregularity in the oscillations or period-doubling bifurcations. To illustrate, the Mackey-Glass equations have shown that variations of chemical concentrations, such as the production rates of proteins within cells or the cell-density variation, may exhibit a time-delay (82). An increase in production rate k caused by a time-delay *k*(*t* — *τ*) may result in pathological diseases. Time-delays are control parameters which above a certain value may result in chaotic cellular oscillations (82). According to Jensen et al. (18) a negative feedback loop is a sufficient requirement for chaotic behavior to emerge within cell signaling. A negative-feedback loop ensures the presence of a time-delay in cellular oscillations, and thereby may act as precursor for the onset of chaotic dynamics (18).

There are also theoretical works by Kaneko and Furusawa exploring the cancer stem cell hypothesis through the lens of cell adhesion and oscillatory nonlinear dynamics which warrant further investigation (98–100). In their chaos hypothesis, they propose that the robustness and differentiation of stem cells towards their multipotent complex cellular states can be predicted by chaotic intra-cellular chemical dynamics (98). Intracellular chaotic oscillations were suggested as markers of pluripotency (stemness) (100). Dysregulated focal adhesion dynamics to the extracellular matrices are hallmarks of cancer metastasis and EMT state-transitions/plasticity dynamics. Most cancer-related deaths are caused by metastatic invasion, and hence elucidating the nonlinear dynamics underlying such plasticity transitions/differentiation dynamics may help identify causal markers in controlling and regulating their behavioral patterns.

While mathematical models, such as differential equations with time-delays, may in principle capture strange attractors within cellular protein flows and gene signaling, experimental confirmation of intracellular chaos remains a fundamental roadblock in complex systems research. Therefore, the quantification of protein oscillations, using the techniques described above must be performed in cancer cells and (CSCs) in time-series and subjected to the various behavioral detection methods enlisted herein. For instance, we can identify the negative feedback loops regulating the Suvà glioma stemness network (*POU3F2*, *SOX2*, *SALL2*, and OLIG2) and experimentally quantify their oscillations using time-lapse fluorescent-imaging within GBM-derived cancer stem cells to understand their cell fate dynamics and reprogrammability (71). Without such experimental datasets, the plausibility of chaotic dynamics as a hallmark of cancer signaling/progression cannot be verified and computational/systems oncology will remain bound to computational and mathematical models. Having laid the basic intuition for cancer stemness and the complex feedback loops regulating their oscillatory dynamics, the following methods are discussed as approaches to detecting chaotic behavior and complex dynamics in cancer networks given their time-series signals are made available.

## Takens’ Theorem

Let us suppose the flows of protein densities and gene expression dynamics of cancer (stem) cells are available in time-series, and a normalized gene expression (or protein oscillation) matrix, array of genes (proteins) by cells with their count, is produced for each time-point. Takens’ time-delay coordinate embedding is the state-of-the-art approach for the attractor reconstruction underlying these complex signals. In 1981, Takens demonstrated in his embedding theorem that the topological dynamics of a complex multidimensional system can be derived from the time-series of a single observable variable (101). The embedding theorem was first demonstrated in the study of fluid turbulence. The term *strange attractors* was coined by Ruelle and Takens to describe the multifractal patterns observed in the bifurcations of turbulent fluid flows (3, 102). They defined a strange attractor as the local product of a Cantor set and a piece of a two-dimensional manifold (102). Takens’ theorem can be used to embed the three-dimensional flows of not only turbulent flows but in principle, any chaotic signal in state-space.

According to Takens’ theorem, you can roughly reconstruct the state-space of a dynamical system by delay-embedding only one of its time-series projections, given the assumption that the variable X contains redundant information about Y and Z variables (i.e., they are causally- related) (See rEDM link in the **Appendix**). From the perspective of Shannon’s information theory, the optimal time-delay *τ* to reconstruct the state-space attractor would correspond to the minimum Mutual Information (MI) of the system (103). The complex structure obtained by the embedding is the *attractor*. As discussed, in chaotic systems, an attractor with a (multi) fractal dimension is observed, known as the strange attractor to which the trajectories of the chaotic system are bound to. Thus, once an attractor is obtained from the time-series embedding, the fractal dimension and Lyapunov exponents can be computed to assess the stability of the dynamical system and verify if the identified attractor is a *strange attractor*. This time-delay embedding procedure scales with the time-series. The longer the time-series, the larger the network and the more complex the attractor obtained. Perturbation analysis can assess the stability and robustness of the complex attractor and provides a powerful toolkit for complex networks analyses in Algorithmic Information Dynamics (AID), as will be discussed later.

An rEDM package for time-delay embedding on experimental time-series is provided in the **Appendix**. rEDM is an R-package for Empirical Dynamic Modelling and Convergent Cross Mapping (CCM) as devised by Sugihara et al. (104). The causal relationships in complex disease signaling networks can be identified using CCM (105). The rEDM package uses a nearest neighbor forecasting method with a Simplex Projection, to produce forecasts of the time-series as the correlation between observed and predicted values are computed (104). CCM is an embedding technique which combines Takens’ theorem and Whitney’s embedding theorem. CCM measures the extent to which states of variable Y(t) and Z(t) can reliably estimate states of variable X(t), as explained above. This happens only if X(t) is causally influencing Y(t) and Z(t). There is a simpler R-package called ‘multispatialCCM’ (multi-spatial Convergent Cross Mapping), an adaptation of CCM, for chaotic time-series attractor reconstruction available, as well. CCM (Takens’ theorem) should be amidst the first set of causality-inference algorithms used to embed the time-series cancer signals in state-space and reconstruct their underlying attractors. However, there may be dimensionality limits for such approaches when dealing with multi-dimensional complex systems like the cancer transcriptome. These embedding methods may be useful for trajectory inference in cell population dynamics or for brute-force approaches in cellular signaling. For instance, we can embed the time-traces of proteins or signals with predicted chaotic dynamics from literature analysis. Otherwise, the task may be too complex for these methods, and non-traditional detection tools must be employed (i.e., machine intelligence).

## Lyapunov Exponents

Once the chaotic signal has been time-embedded and its attractor(s) has been reconstructed in state-space, quantitative-behavioural detection algorithms can be used to determine if the identified attractor is indeed chaotic (strange). As such, Lyapunov exponents and fractal dimension estimates remain the most robust classical measures of chaotic behavior detection. Positive Lyapunov exponent(s) are robust signatures of chaos quantifying *sensitive dependence on initial conditions*. Prior to measuring the Lyapunov exponents or fractal dimension on the identified attractor, de-noising and filtering algorithms can be used as a pre-processing step to reduce the noise and data dispersion. The intuition behind this is to consider nearby points in the phase space generated by time-embedding and then perturbing each point proportional to a weighted average of the nearby points. Using this, one can recover the fine structure of the attractor in higher dimensions, especially if this is combined with signal smoothening methods (e.g., imputation algorithms).

To illustrate Lyapunov exponents, consider two points of a cancer signal’s trajectory (e.g., a single protein flow or gene expression time-trace) separated by a very small distance in time, x(t), in phase space, then the separation (bifurcation) of its trajectory from its initial position is given by:


$$\delta x\left(t\right)\approx x_{0}e^{{\text{λ}}_{L}\text{t}}$$

for a small time, t, where the *λ_L_* is the Lyapunov exponent. In multi-dimensional dynamical systems, there may be a spectrum of Lyapunov exponents to consider. The Lyapunov exponent measures how far two initially close by points on a dynamical system’s trajectory separate (bifurcate) in time. If the Maximal Lyapunov exponent *λ_L_* > 0 (positive), there may be a chaotic attractor (15). That is, in a chaotic system, the trajectories exponentially diverge apart from each other. In hyperchaotic systems, at least two positive Lyapunov exponents are observed (106). The inverse of the positive Lyapunov exponent $\frac{1}{{\text{λ}}_{L}{+}^{}}\sim \tau_{L}$ denotes the Lyapunov time *τ_L_*, the finite predictability horizon of a chaotic system. However, unlike a random (stochastic) system, the exponentially diverging trajectories will map onto a finite fractal structure in phase space: the *strange attractor*. As mentioned, the fractal topology of chaotic attractors provides the explanation for this counter-intuitive pattern. The fractal topology allows the *stretching and folding* of phase-space analogous to making taffy candies (3). The NP-hard question then is how to find these taffies in the state-space of a given complex system/network’s state-space?

Lyapunov exponents have been used in mathematical and numerical simulation models of cancer population dynamics to verify the presence of chaotic attractors. For example, Itik and Banks (89) demonstrated using a set of ordinary differential equations (ODE) to model the tumor-immune-host cell density dynamics the emergence of chaotic attractors at certain critical parameters such as the growth rate. The existence of chaotic behavior was confirmed by calculating the Lyapunov exponents and the fractal dimension of the observed attractor, which was found to be near that of the Lorenz attractor’s fractal dimension. Thus, the pairing of computational models and simulations with empirical dynamics is fundamental to complex systems research. Chaotic dynamics were confirmed in the model by a positive maximum Lyapunov exponent. As the *α* parameter increased, transition to chaotic behavior was observed in the bifurcation plot by period-doubling cascades, signatures of chaotic dynamics (89).

More recent mathematical modelling of tumor-immune cell predator-prey dynamics have been performed with time-delay as a bifurcation parameter (90). Chaotic attractors emerged in the system’s phase-space and were suggested as indicators of aggressive metastatic transition in cancer cells (89, 90). However, as stated, the lack of time-series experimental datasets remains a roadblock in experimentally confirming the presence of these chaotic attractors in cancer-immune-host dynamics. As such, a python and MATLAB code for Lyapunov exponents calculation from time-series is provided in the appendix to encourage its applications in medical systems. The ‘nolds’ package in python, a small numpy based library, also provides various measures of nonlinear dynamics such as the Lyapunov exponent and Hurst index (see **Appendix**).

## Fractal Dimension and Multifractal Analysis

Fractals are ubiquitous in nature. They are universal patterns exhibiting self-similarity which iterate themselves across many scales (i.e., power law scaling). We tend to think of trees, snowflakes, clouds, blood vessels, bronchi, neural networks, or the coastlines of geographic landscapes when thinking of fractal structures (107). They demonstrate that complex geometric patterns can spontaneously emerge from simple recursive rules. Chaotic attractors and many complex systems exhibit (multi)fractal scaling. A such, fractals serve as a robust measure of both, complexity, and chaotic dynamics. However, there is a caveat. The complexity we refer to here is not algorithmic complexity, but rather complex dynamics and irregularity. From the viewpoint of algorithmic complexity, only a very short program is required to generate fractal patterns. Hence, there is some ambiguity between complex fractal dynamics and algorithmic complexity measures, in this domain of research. Fractal geometry explains the paradox of how strange attractors compactify infinite curves into a finite space (area or volume), and as such we may define this recursive irreducibility as complex dynamic structures.

The word fractal, coined by Mandelbrot, is derived from the Latin word *fractus* meaning *fragmented*. Formally, a fractal is defined as a mathematical object with a fractional (non-integer) dimension (107). Gaston Julia first demonstrated that iterated functions of complex numbers can generate fractal patterns. However, Mandelbrot computationally generated fractals and demonstrated their universality in Nature and chaotic systems (107). The simplest example of a fractal is the Cantor Set (dust) in which one starts with a straight line and as we keep removing the middle one-third of the line with each iteration, the fractal is generated. Another set of examples are the Serpinski’s gasket and Koch’s snowflake, both generated by simple recursive rules starting from an equilateral triangle. Fractals can also be continuous. A good example would be Hilbert’s space-filling curves which remind us of Escher’s tessellations or the honey-comb lattices in beehives. However, the most popular example of a fractal is the Mandelbrot Set, the set of all Julia sets, described by the iterative complex function z defined at n-iterations by the rule: *z_n_*_+1_ = *z_n_* + *c*, where the complex number c is its initial condition *z*_0_. The Mandelbrot set demonstrates a central property of many complex systems, that complex structures and patterns can be generated from simple, recursive feedback loops.

Fractals are some of Nature’s most stable structures demonstrating the optimization of space (compactification) and its spatial resources. A system exhibiting fractal architecture is robust to environmental changes. For example, bees use a fractal space-filling architecture, composed of hexagonal symmetry to optimize the area: curve length (perimeter) ratio in building their beehive structures. The same principles of hierarchical spatial organization and resource optimization may apply to Nature’s intelligent exploitation of fractal geometry, as an adaptive strategy to minimize the amounts of resources used and wastes produced by a complex system (107). For instance, oil spills exhibit fractal patterns in ocean floors and lakes (108). The fractal dimension can be computed from their imaging power spectra (the ratio between powers at different scales) to characterize their texture analysis (108). Their fractal structure may imply that they are difficult to treat as their patterns and information repeatedly span across many scales. Similarly, studies have shown that tumor textures can be characterized as multifractal structures (109–111). In the study of tumor structures, fractals have been restricted to describing the self-similarity of abnormal blood vessels (angiogenesis) and tumor contours across many length scales, as a measure of its spatial roughness (110). However, it can also be applied to time-series analysis as well, as in the case of detecting strange attractors from complex signals. The multifractality of tumors may reveal their aggressiveness, resilience (to environmental perturbations) and hence, be indicators of tumor relapse and therapy resistance (89). Mesoscopic mathematical models of tumor pattern formation dynamics have also suggested that a fractal dimension analysis could provide a quantitative measure of its growth forecasting and irregularities (112, 113).

The Fractal Dimension (FD) is a statistical measure of complexity which occupies a fractional dimension, in between two consecutive integers. That is, if a point has a dimension of zero, a line is 1-D, a plane is 2-dimension and volume is 3-D, fractals occupy dimensions in between these integers. FDs can be used to characterize the structural complexity (roughness) of tumors and their irregularity in their signaling dynamics (time-traces). For example, the tumor vasculature was shown to have a higher fractal dimension of 1.89 ± 0.04, whereas normal arteries and veins yield dimensions of 1.70 ±0.03 (109, 110). The higher FD indicates an increased roughness and complexity of the vasculature. The fractal dimension of an image, such as medical imaging of tumor structures, may be estimated by various techniques: (*a*) box-counting/cube-counting (for volumetric systems); (*b*) correlation; (*c*) sandbox; (*d*) Fourier spectrum, etc. When applied to images of blood vessels, these methods yield scaling relationships that are statistical best fits to a power-law relationship within a finite range of scales. Although, these fractal dimension estimation algorithms differ, they obey to the same calculation basis summarized by the three steps: (1) Measure the quantities of the object using various step sizes, (2) Plot log (measured quantities) versus log (step sizes) and fit a least-squares regression line through the data points, and (3) Estimate FD as the slope of the regression line (15, 114).

The most widely used FD computing algorithm is the Box count algorithm. In the Box counting method the signals are represented on a finite scale grid and the grid effects interplayed with the computing fractal dimension. The box-counting method asks: How many boxes are needed to cover the fractal? A fractal can be described by a power law scaling given by *N*∝*r* ^−*D*^ where N is the number of boxes needed to cover the object/pattern, r is the length of the box, and D is the fractal dimension. Then, $=\frac{logN}{\text{log}\frac{1}{r}}$ ,where 1/r is the inverse of the box size r (114). Therefore, the slope of the log-log plot of N and r is the Fractal dimension. However, what if the system requires more than one FD to characterize its statistical patterns? Complex dynamical systems may exhibit multifractality when there are scaling processes in time. The time-series power (frequency) spectra of fluid turbulence exhibit intermittency and fluctuations, which necessitate the use of multifractal analysis (57). There may be hidden spikes (sudden transitions) in the intermittent fluctuations of experimental fluid turbulence (57). Multifractal analysis provides a powerful tool to characterize these fluctuations in complex dynamical systems.

Multifractal analysis was first introduced by Mandelbrot in the study of turbulence-mediated flow velocity patterns. The Multifractal spectrum can be quantified by the following descriptors: (a) the Hurst exponent, (b) the slope of the distribution produced by the collection of the Hölder regularity index *α*, and (c) the width spread (broadness) of the spectrum, characterizing the variability of the Hölder exponents (115). The local Holder exponent *α* is a local measure of roughness, and an exponent of a power law characterizing the multifractality of the system. As the word *spectrum* implies a multifractal is a process exhibiting scaling for a range of different power laws. Various methods exist for computing the multifractal spectrum of Hölder exponents (i.e., the slope of the log-log plot of the power law system), which include fractional Brownian motion (fBm) methods, and the most popular are the wavelet-transform based methods. For instance, the Wavelet Transform Modulus Maxima (WTMM) method, uses the continuous wavelet transform to compute the Hölder exponents. It is more efficient than the box-counting algorithm. The local Holder exponent is defined as:


$$d_{loc}\left(x,y\right)=\underset{n\to \infty }{\lim}\frac{\log\left(Prob\left(i_{1}\ldots i_{n}\right)\right)}{\text{log}\left(2^{-n}\right)}$$

Where the Prob (…) term is the probability that the point (x,y) of the signal lies in a square with indices (i_1_…i_n_). N addresses the number of squares containing (x,y). As we take the limit defined, we get the multifractal spectra a collection of all points of the fractal having the local Hölder exponents alpha (i.e., d_loc_ becomes alpha) (116). The Hurst index, H, describes the roughness of the time-series. It takes a value in between 0 and 1 wherein H= 0.5 denotes a true random process (i.e., Brownian time-series). The smaller the value of H, the higher the roughness, and vice versa.

## Criticality

A feature of many robust complex systems is *criticality* (i.e., edge of chaos). The transition from criticality to chaotic dynamics may be most useful for our discussion of cancer cell fate dynamics. Critical systems are a class of nonequilibrium systems exhibiting scale-invariant spatial-organization and scale-free dynamics (117). In nonequilibrium systems, the critical points indicate regions where the attractors governing its phase-space dynamics are located (118). It has long been suggested by Kauffman et al. that gene regulatory networks operate in the critical phase between regularity and chaos. Critical dynamics of the network were suggested to permit the coexistence of robustness and adaptability/resilience in cellular systems, thereby allowing both the stability of cell fates and their epigenetic switching between multiple phenotypes (network states) in response to environmental fluctuations/perturbations and/or developmental cues (119). The emergence of phenotypic plasticity may be deep-rooted in critical dynamics in complex networks and chromatin states configuration. However, the lack of time-series datasets limits the observations of critical network dynamics and critical cell-state transitions to computational models such as Boolean networks (120). Further, the lack of time-series data points has fundamentally limited the investigation of critical dynamics in cellular processes to simulations. Therefore, simulations-driven artificial intelligence (AI) is a powerful platform in complex systems research granting us insights into otherwise intractable problems.

In dynamics, a critical point or tipping point is a state that lies at the boundary between two phases or regions that have differing behaviors and rules. According to Renormalization Group theory, phase transitions are characterized by a divergence (i.e., tends to infinity) in the coherence length (the characteristic length scale) of a system near some critical point (55). The corresponding quantity of interest in a dynamical system is the correlation time, the length of time for which perturbations are propagated into the future. Critical systems exhibit *power law* behaviors as signatures of their long-range interactions in the system. Criticality may be an indicator a complex system’s potential to *transition to chaotic behavior* (53, 55). As such, the detection of power law statistics can be indicators of complex dynamics, and studying criticality reduces the search space of potential chaotic oscillators within a complex network.

Critical dynamics have been modelled in the cell fate transitions (differentiation dynamics) from healthy to cancer cell states (72). Rockne et al. (72) demonstrated the state-transition dynamics from healthy peripheral mononuclear blood cells (PBMC) to acute myeloid leukemia (AML) in mice can be described by a double-well potential with two critical points. They demonstrated the critical points in the transcriptomic state-space can predict the cell fate trajectories during disease progression. The 2D transcriptomic state-space was obtained from dimensionality reduction analysis (PCA- principal component analysis) on the time-series bulk RNA-seq data (72). The transcriptome was modelled as a particle undergoing Brownian motion using the Langevin equation in a double-well quasi-potential U(x) with two stable states, the critical points, representing the healthy and AML states, respectively. To calculate the mean expected stochastic behavior of the cells, they considered every point in the transcriptome state-space as a particle characterizing a cell. The evolution of the probability density function of all such particles (cells) was obtained by the Fokker–Planck equation (FPE) (72). Although, the model beautifully illustrates critical dynamics in cancer cell fate transitions, the assumption that the healthy state and AML state critical points are *stable phenotypes* may be the issue with the model. The cell states were assumed to be *stable fixed-point attractors* separated by an unstable transition-state higher in potential energy. As discussed herein, cell phenotypes may correspond to various types of attractor patterns (i.e., fixed-points, limit cycles, tori, chaotic) on the multidimensional signaling state-space. However, one-dimensional dynamical systems, as modelled in this study and most studies in systems oncology, are limited to fixed-point attractors only.

Cell fate bifurcations may exhibit critical dynamics. A key mechanism for cell fate transitions in cancer systems is the EMT program (Epithelial-Mesenchymal Transition) and remains as one of the primary examples of critical cell fate dynamics observed in the computational models by Nieto-Villar et al. and Jolly et al. EMT programs govern many cancer-related behaviors such as the transition from one cancer phenotype to another, stem cell plasticity, cancer metastasis and chemoresistance. EMT switches are essential in the critical dynamics of CSCs and non-CSC phenotypic switching (20; 97).

EMT transitions have been modelled as first-order phase -transitions, a signature of critical systems, in computational tumor dynamics models by Guerra et al. (121). Cancer evolves along three basic steps: avascular, vascular, and metastatic, all emerging downstream of biological phase transitions. Guerra et al. (121) demonstrated a network model of EMT dynamics consisting of four interacting cell types, wherein N represented the population of normal cells exposed to pro-carcinogenic stimulus, H the healthy cell population (mainly epithelial phenotype), and M is the population of mesenchymal cells (121). The immune population I was used as the control parameter (can fluctuate). The network consisted of various cellular processes including mitosis and apoptosis of the proliferating tumor cells. Mathematical models of chemical kinetics were used to reduce the network to a system of ODEs representing the EMT dynamics. Lempel-Ziv compression algorithm, Lyapunov exponents, and the Lyapunov fractal dimension were assessed on the computational model dynamics. At some threshold of the control parameter I, EMT was observed in the dynamics as characterized by a supercritical Andronov-Hopf bifurcation and emergence of a limit cycle (121). As the control parameter I further decreased below critical thresholds, complex Shilnikov-bifurcations were observed, and the population dynamics eventually gave birth to chaotic dynamics. The computational model exhibited that under decreased immune dynamics (i.e., lower I value), the tumor cells exhibited apparently random behavior (i.e., chaotic dynamics), thus promoting mesenchymal phenotypes as indicated by the EMT phase-transition (121).

Further in evidence to critical dynamics in EMT systems, one of the many complex signals mediating EMT transition is the microRNA-200/ZEB mutual inhibitor feedback loop, driven by the transcription factor SNAIL (122). A simulation-based study found that mRNA levels of ZEB can indicate the critical tipping point for EMT phase transitions in cancer cells (122). An increased variance, autocorrelation, and conditional heteroskedasticity were shown to dynamically vary during the phenotypic transitions, with an increased attractor basin stability observed for the hybrid EMT state, indicating it may be the fittest phenotype for metastatic progression (122). The mathematical models by Sarkar et al. showed a cusp-like catastrophe in the EMT plasticity bifurcation diagram, suggestive of a critical phase-transition.

Sarkar et al. (122) considered a mathematical model of microRNA-based chimeric circuit capturing the binding/unbinding catalytic kinetics of associated protein complexes and transcriptional machineries. Given m is the abundance (number) of mRNA, let n be the abundance of microRNA, and B be the TF protein of interest, then, we have the first-order kinetic equations:


$$\frac{dn}{dt}=g_{n}-mY_{n}-k_{n}n$$

$$\frac{dm}{dt}=g_{m}-mY_{m}-k_{m}m$$

$$\frac{dB}{dt}=g_{B}mL-k_{B}B$$

Where g corresponds to the synthesis rates of the respective molecules in subscript, and in particular, *g_B_* is the translation rate of protein B for each m in the absence of n. The k parameters denote the degradation rates of the molecules, Y and L are the n-dependent functions denoting the various effects of miR-mediated repression (122). These differential equations were computationally simulated using Monte Carlo simulation in which each reaction event is considered as a Markov process (122). The time and species numbers were updated stochastically by choosing a random reaction event. The miR-200 based chimeric tristable miR-200/ZEB circuit was simulated by casting 10 reaction events as a function of the number of SNAIL molecules. The corresponding Master Equation was simulated with the Gillespie algorithm to obtain the stochastic trajectories from which the critical transitions was identified with a cusp-like phase-transition in the bifurcation plot (122, 123) have extended the understanding of these EMT switches by use of network approaches to investigate cellular decision-making in EMT phenotypic transitions and how they regulate emergent behaviors such as phenotypic plasticity dynamics (i.e., the ability to reversibly switch/transition in between heterogeneous phenotypes) in tumor ecosystems. The study shows that network topology influences phenotype commitments and canalization signatures of the tumor differentiation/developmental landscape. Many other mathematical studies demonstrating phase-transitions and complex dynamics in metabolic tumor growth models with glycolytic oscillations have been established by the Nieto-Villar group, which shall not be discussed herein (124–126).

The above-listed studies show that computational simulations/modelling paired with complex networks analysis pave fruitful insights into many other complex cancer processes. There are various other tools borrowed from nonequilibrium statistical physics one can use to investigate criticality and phase-transitions in complex systems. The breadth of this topic deserves a separate paper of its own and cannot be confined to this brief survey. However, the gist of these approaches are summarized below by two of three key techniques that may be useful for cancer research: percolation clustering, the Ising spin glass model, and Cellular Automata (CA).

Cancer networks can be visualized as Boolean networks, in which the elements of the networks, such as gene expression, can either be on or off, described by 1 and 0, Boolean states. This allows them to be ideal models for the Ising model adaptation, where the Boolean states correspond to gene-type spins (spin up and spin down). Further, many complex cancer processes including phosphoproteomics (on/off switches of protein conformations) and epigenetic-chromatin states (e.g., acetylation/methylation dynamics of histones) can be defined as binary states. As such, Ising models are simple yet powerful tools to study cell fate transitions from such complex gene and protein network dynamics (54, 127). However, even the optimization of a two-dimensional Ising model is NP-hard. Therefore, mean-field theory approximation or iterative random searching algorithms such as Monte-Carlo methods (e.g., the Metropolis algorithm) are used to find approximate solutions. For example, Lang et al. (128) using single-cell data demonstrated the rugged energy landscapes of Ising models can be used to visualize/reconstruct the distinct cell states from transcriptomic data as attractors of the Waddington epigenetic landscape (128). The flipping of spins caused the step by a step-change in the phenotype of the cell, mapping cell fate transitions from one attractor to another. Ising models can predict the co-existence of structurally or functionally organized clusters in complex networks, as well. Ising models also serve as the theoretical framework of Artificial Neural Networks such as the Hopfield neural networks (129). Hopfield networks are emerging as machine learning approaches for causal inference in complex multiscale cellular dynamics including classifying or predicting gene expression patterns and forecasting their epigenetic landscapes (130).

One of the issues in the understanding of critical dynamics, such as EMT processes in cancer cells, is the lack of a mathematical theory/mechanisms to explain their transition to chaos. The works by Kauffman et al. in this regard have only been qualitative for the most part (119). On a tangential note, in literature, one usually distinguishes chaos from “order.” This phrasing is extremely common in complex systems research, but it is technically ambiguous. In the 1960s, Prigogine, a pioneer of complexity, demonstrated that disordered, far-from equilibrium chemical systems can spontaneously give birth to orderly stable states (i.e., dissipative structures) (131). Prigogine defines the self-organization of these dissipative structures as *order out of chaos*. Therefore, *order* may be an emergent behavior of chaotic systems. As such, it is technically more accurate to distinguish chaos from “regularity” or periodicity, rather than “order.”

Cellular Automata (CA) are discrete dynamical systems, consisting of a grid/lattice of adjacent cells updated by simple local rules. As mentioned, the Bak sandpile model was a CA which showed self-organized critical dynamics and emergent patterns of behaviors. As such, CA are versatile tools for modelling complex and critical systems (56, 132). Further, powerful complex systems frameworks such as tools from Algorithmic Information Dynamics can be coupled with CA to study complex networks dynamics and biological pattern formation (133).

CA are spatiotemporally discrete patterning systems represented by lattices of local interactions. The transition rules are local and only depend on the site neighborhood interactions on the lattice. Traditionally, at every time step, every lattice suite updates its state simultaneously. However, there are variants of CA such as asynchronous CA and/or inhomogeneous CA where such rules do not apply, as seen in tumor growth models (134). There is a vast amount of literature on the use of CA to model tumor growth dynamics. Some examples include avascular tumor growth models with the CA system modelling reaction-diffusion equations, partial differential equations (PDE) modelling the tumor-immune-host cell dynamics in varying nutrient conditions (135). Similar works were seen with inclusion of the effects of chemotherapeutic drugs on the tumor growth in reaction-diffusion PDE models by Ferreira et al. (136) and in the ODE models of de Pillis and Radunskaya (137).

In a typical avascular tumor growth model, we have a regular lattice wherein the discrete states correspond to biological (Cancer) cells. If a cell dies, the lattice is unoccupied (134). A cell can survive, divide, or die, and local rules can be updated asynchronously. We can also simulate the ecological dynamics or competitive interactions between multiple cell types, as seen in heterogeneous tumor microenvironments (134). For instance, the hybrid PDE-CA simulations by Mallet and Pillis (135) closely match the population dynamics observed in experimental tumor-immune dynamics. The models can also simulate complex tumor processes such as immune-cell filtration and tumor immune escape (135). The nutrient species’ reaction-diffusion dynamics govern the tumor growth model in these CA systems. Let us consider a simple model system with two nutrients, then the reaction-diffusion system is given by:


$$\frac{\partial N}{\partial t}=D_{N}\nabla^{2}N-k_{1}HN-k_{2}TN-k_{3}IN$$

$$\frac{\partial M}{\partial t}=D_{M}\nabla^{2}N-k_{4}HM-k_{5}TM-k_{6}IM$$

Where, M and N represent the proliferation nutrient and survival nutrient concentrations, respectively (135). The cell species’ abundance are given by H for the host cells, T for the tumor cells, and I for the immune cells. D refers to the diffusion coefficient of the respective nutrient species indicated by their subscript (135). The rate constants k indicate the respective consumption rates for each of the nutrient for each of their assigned cells (H, T, and I). The nutrients can also represent activators, inhibitors, or other protein complexes (e.g., enzymes, epigenetic modulators, drugs, chemical exposures/carcinogens, therapies/perturbations, etc.) as chosen appropriate for the model system of interest.

Further, CA such as Conway’s Game of Life can stochastically simulate cancer cell kinetics and their multi-scale tumor population dynamics (138). CA are thus tools for computational systems oncology, to monitor tumor growth dynamics under drug perturbations or targeted therapies, in software space (*in silico*) (139. For example, (140) demonstrated that CA models can simulate the behavioral dynamics of cancer stem cells (CSCs), which as discussed, are believed to be in large part, responsible for the emergent adaptive behaviors in tumor ecosystems. To further illustrate, in another set of studies, stochastic CA models well-captured the dynamics of avascular tumors under chemotherapy and immunotherapy perturbations and provided computational insights into how drug delivery should be optimized to inhibit tumor proliferation (141).

To further illustrate, in a model by Qi et al. (142), a two-dimensional lattice was used with four discrete states, one denoted cancer cells, one represented normal healthy cell, and the other two represented immune cells interacting with the tumor and host cell environments. Probabilistic rules with non-local and non-homogeneous transition rules updated synchronously, resulted in the emergence of cancer cell behaviors which closely matched experimentally observed Gompertz growth models. Similarly, Kansal et al. (143) simulated a brain tumor growth model *via* a three-dimensional Voronoi network, with three discrete states representing three types of malignant cells: proliferating cells, quiescent cells, and necrotic cells. Similar local transition rules and conditions as the model by Qi et al. were then used to model the tumor growth dynamics. The pattern dynamics closely matched those observed in experimental brain tumor data.

A rich repertoire of experimentally validated work on cellular automata-based approaches in tumor modelling has been performed using the Cellular Potts model (CPM). The CPM, also known as the Glazier-Graner-Hogeweg model is a time-discrete Markov chain spatial lattice model for studying complex cellular dynamics in biological populations (144). Some pertinent examples of such complex cellular processes include cell-interactions mediated collective behaviors (e.g., collective cell migration, cell fate decision-making/differentiation dynamics, etc.), and multiscale pattern formation systems including cancer morphogenesis and tumor invasion dynamics (145–148). The individual cells are represented by simply-connected domains on nodes for a given cell index. The CPM dynamics evolves by updating the lattice configuration one cell at a time based on probabilistic transition rules following a modified Hamiltonian-dependent Monte Carlo simulation/Metropolis algorithm (144). The experimental works of Sen’s and Bhat’s groups have well-supported the applications of the CPM model and similar CA-based computational modelling in decoding the complex multicellular dynamics underlying cancer metastasis and invasive- extracellular matrix (ECM) remodelling (149–151). For instance, (151) validated that the reaction-diffusion mediated multiscale focal adhesion dynamics and ECM-remodelling of breast carcinoma can be accurately model the cancer invasion processes.

Other models of multi-cellular tumor growth systems and tumor angiogenesis (i.e., vascular tumors) have also been successfully reproduced using CA systems (134). For instance, in a two-dimensional lattice CA, a square topology with a nine-membered Moore neighborhood was used in a tumor growth model by Serra and Villani (152). The model accurately reproduced *in vitro* tumor cultures’ growth dynamics with varying growth conditions such as the difference after exposure to carcinogens and the resultant development of transformation foci. The model quantified the effects of the chemicals and the change in the culture medium by its exposure to good precision matching those obtained by mean-field theoretic approaches on underlying ordinary differential equations. In principle, these approaches can also be extended to *in vivo* tumors and patient-derived xenografted tumor modelling for monitoring the responses of targeted precision therapies. These are some of the many examples to illustrate that CA are robust tools in quantitative and computational oncology to model tumor growth dynamics under therapy control and help regulate/adjust clinical decision making towards optimized precision medicine.

Criticality, the state of being poised between regularity and chaos, was illustrated as a hallmark characteristic of cancer processes including EMT switches, cell fate plasticity dynamics, tumor pattern formation, metastatic invasion, and complex dynamics in computational epigenetics (e.g., chromatin-epigenetic modifications during cell state transitions). Cellular automata (CA) was discussed as a powerful computational modelling approach to investigate these critical dynamics in multiscale cancer systems.

## Entropy

Entropy is seen as a measure of uncertainty or disorder in traditional branches of physics such as statistical mechanics and thermodynamics, respectively. For instance, a gas of molecules has a higher entropy than its liquid or solid phases because a greater number of rearrangements of its microstates (particles) would correspond to the same macrostate (gas). However, in complex systems theory, (Kolmogorov-Sinai) entropy is discussed as an information-theoretic measuring the flow of information across state-space by the trajectories of a dynamical system. Takens (101) described topological entropy as one of the traditional measures for chaotic-behavior detection in fluid turbulence. The phase-space flows of the system can then be quantified as a transfer of information. The time evolution of the set of orbits originating from all possible initial conditions of the system generates a “flow” in state-space, governed by a set of n first-order differential equations: $\frac{dX_{i}}{dt}=F_{i}\left(x_{1},x_{2},\ldots ,x_{n}\right)$ , where n is the dimensionality of the space and X is the state-vector characterizing the trajectory of the dynamical system. If we can assign probabilities *P_i_* to each of the possible outcomes in the bifurcations of the system, we can define the information associated with the outcome as given by the Shannon’s entropy: *H* = Σ*_i_P_i_log*_2_ *P_i_*. When entropy increases sufficiently high beyond some critical value of the governing parameters, a phase-transition can occur as denoted by the bifurcations of the attractor dynamics (e.g., transition from a fixed-point to a limit cycle or, from an oscillation to chaotic attractor). The dynamical systems analog of Shannon’s entropy is formally referred to as the Kolmogorov-Sinai (KS) entropy or metric entropy. A system with positive Lyapunov exponents will show a positive KS entropy (153). There is also another useful entropy measure for dynamical systems known as topological entropy, a variant of the metric entropy, wherein instead of a probability measure space we use a metric space with a continuous transformation. Their uses may depend on whether we are dealing with ergodic, flow preserving systems.

The onset of phase-transitions can be quantitatively measured using an information production rate given by the entropy rate: dH/dt. In chaotic systems, an increasing (positive) entropy rate is observed. Intuitively, the increased entropy rate can be interpreted as a measure of unpredictability and irreversibility in the information flow of the system. Thus, maximal entropy and positive entropy rates can be used as predictors of the birth of complex attractors (i.e., strange attractors) in the phase-space of the dynamical system. However, some works have established that entropy is not a robust measure of complexity. The Shannon entropy fails to capture the algorithmic content of a dataset and thereby fails as a measure of graph (network) complexity (154). The KS entropy (rate) can quantify the amount of information (flow) from or within system but whether the information flow is causally related or not cannot be inferred. Regardless, they can be used as cross-validation techniques for chaos detection and may be useful for Waddington landscape reconstruction (i.e., quantify cell state attractors using metric entropy measures).

## Simulations and Computational Dynamics

Due to the limited availability of three-dimensional time-series datasets, the study of complex dynamics within cellular (cancer) cybernetics heavily depends on computational simulations. Computational simulations are emerging as powerful tools for reconstructing chaotic attractors and inferring chaotic or critical dynamics in biological networks. As discussed, Sarkar et al. (122) used simulations of differential equations to infer critical dynamics in simplified cancer-EMT networks. We also discussed the appearance of chaotic oscillations in cancer-immune competitive growth dynamics when time-delay was introduced as a control parameter in simple modelling differential equations (90). The emergence of chaotic attractors was suggested as indicators of long-term cancer relapse and the emergence of aggressive cancer phenotypes (90). Let us consider an example from the works by Jensen et al. on the use of simulations to detect intracellular chaos in protein oscillations. Their works are an extension of the Goodwin oscillator model (81) to cancer-relevant protein systems.

A Transcription Factor (TF) is a protein which binds to the enhancer or promoter regions of a gene of interest with some affinity and forms a complex with RNA polymerase to transcribe the gene. The control of transcription regulates gene expression and its resultant protein translations. Many cancer-related TFs exhibits oscillatory dynamics within cells (18). For instance, the oscillations of the tumor suppressor p53, Wnt, and NF-kB are TFs central to regulating immune response, apoptosis tumorigenesis, and cancer cell division. The works by Jensen et al. (18) have shown that oscillatory external stimuli might induce chaos and phase (mode)-locking inside cells when coupled to their internal protein oscillations. Using microfluidic cell cultures, Heltberg et al. (155) delivered periodic TNF simulation to fibroblasts and recorded the NF-kB nuclear localization by live cell fluorescence imaging. CellProfiler and MATLAB peak analysis algorithms were used to track cells and quantify the NF-kB translocation, where the activation was quantified as mean nuclear fluorescence intensity normalized by mean cytoplasm intensity (155). The phase locking transitions in an oscillatory manner were observed even amidst noise fluctuations at critical bifurcation points. When the oscillations entered a chaotic state, counter-intuitively, the NF-kB protein was shown to be most effective at activating downstream genes and optimizing their signaling cascades (19).

The emergence of intracellular chaotic behavior, at the level of protein oscillations, remains highly controversial and subjected to debate. However, the computational simulations paired with experimental models, as performed by Jensen et al. (18) are the first set of studies showing *deterministic chaos* can drive a nonlinear internal oscillator within cells such as NF-kB with a periodic external signal such as the cytokine TNF. With low level amplitude oscillations of the external driving signal, it can entrain or synchronize with the nonlinear oscillator as indicated by the Arnold tongues observed in its bifurcation diagram (19). Arnold tongues are regions of parameter space where the NF-kB oscillations are entrained to the external TNF oscillation. Entrainment implies frequency and phase-locking. Outside the Arnold tongue, there is no synchronization. As the TNF amplitude increases beyond a critical threshold, chaotic dynamics can occur as indicated by period-doubling bifurcations and the overlapping of Arnold tongues (19). The model shown by Heltberg et al. consisted of a negative feedback loop system (with inhibitor IkB*α*) in a single nonlinear oscillator (19). These simulations show that the strong coupling of two nonlinear oscillators with a negative feedback loop can give rise to complex dynamics in cell states. These findings suggest that a negative feedback loop in protein oscillatory networks may be a sufficient condition for driving chaos in cells.

Lastly, we will discuss simulations and computational modelling in epigenetics as an example of multiscale dynamics in computational/systems medicine. *Computational epigenetics* is a field at its infancy in comparison to simulations of cancer cell population dynamics or patterns of regulatory network dynamics. As discussed, forecasting the long-range interactions in 3D-genome structure, histone interactions, and other epigenetic processes is the key to deciphering cancer stemness networks and phenotypic plasticity in cancer cell fate dynamics and commitments. These emergent behavioral patterns are the drivers of adaptive features in cancer ecosystems such as therapy resistance and intratumoral heterogeneity. One of the best examples of epigenetic control and regulation in tumor transcriptional dynamics is the polycomb memory system in pediatric high-grade glioma.

Current approaches to modelling histone mark spreading dynamics and chromatin looping dynamics include ordinary differential equations (ODEs) modelling the catalytic kinetics with their (experimentally confirmed) rate constants, or stochastic kinetic models., the latter of which remains the most widely employed approach due to the analytical constraints of ODEs. We can consider a simple epigenetic feedback circuit like the antagonistic feedback between H3K27 and H3K36 methylation (156), or a much simpler single histone modification’s methylation or acetylation dynamics for such ODE models followed by some iterative differential equation solver to approximate the solutions (e.g., Euler methods, Runge-Kutta, etc.) (157). In the case of more complex, scalable models, like the antagonistic H3K27me2/3 and H3K36me2 circuit, ODE simulation toolkits such as AMICI (combines SUNDIALS and SuiteSparse) and PESTO are available for ODE solving and gradient-based parameter estimation (156). To illustrate, the methylation dynamics of the histone mark H3K79me0/1/2/3 by the enzyme DOT1L (known for impaired functions in leukemias), can be given by the following system of ODEs:


$$\frac{d\left[me0\right]}{dt}=-k_{on}\left[me0\right]+k_{off}\left[me1\right]$$

$$\frac{d\left[me1\right]}{dt}=k_{on}\left[me0\right]-k_{off}\left[me1\right]-k_{on}\left[me1\right]+k_{off}\left[me2\right]$$

$$\frac{d\left[me2\right]}{dt}=k_{on}\left[me1\right]-k_{off}\left[me2\right]-k_{on}\left[me2\right]+k_{off}\left[me3\right]$$

$$\frac{d\left[me3\right]}{dt}=k_{on}\left[me2\right]-k_{off}\left[me3\right]$$

Where t denoted time, *k_on_* is the forward methylation reaction rate and *k_off_* is the reverse reaction rate (also accounts for cell division, nucleosome turnover, and demethylation) (157). The brackets [] denote the concentration of the specific histone methylation marks, where 0 is unmodified H3K79 and 3 refers to the trimethylation.

In contrast, the more popular set of approaches in epigenetic modelling of chromatin or histone state dynamics involve stochastic models such as Monte Carlo simulations, Langevin dynamics/Random walks, and coarse-grained molecular dynamics. For instance, a recent study has shown that stochastic computational simulations can well predict PRC2 dynamics in glioma systems, even under the presence of antagonistic H3K36 modifications and H3K9me3 marks propagation dynamics (31). The stochastic simulation STOPHIM adopts a bi-modal random walk model of PRC2-mediated histone methylation dynamics across a simulated genomic region represented as a 1D-vector. The study found that H3K27me 2/3 marks which are widely deposited by PRC2 across broad genomic regions, show globally inhibited methylation distribution patterns in H3K27M glioma cells (31, 158). Although, the model includes cooperativity in PRC2 dynamics, and the simulated kinetics/catalysis of the methylation rates agree with experimental data, chromatin phase-separation and 3D-genomic structural organization is lacking due to the adopted 1D- linear model. The integration of ChIP-Seq and Hi-C data is required to model the 3D conformation dynamics, which is an essential step to forecast critical dynamics (phase-transitions) in histone marks or chromatin states. The next mission for AlphaFold-like algorithms should be chromatin folding and inferring transcriptional states/dynamics from chromatin/epigenetic states.

There are other stochastic/probabilistic simulation approaches available in modelling epigenetic states and histone mark spreading dynamics in cancer systems. Some examples include the use of coarse-grained molecular dynamics from chemical master equations over 1D- lattice models with mean-field approaches (for analytic solutions) used for sirtuin-2 mediated acetylation dynamics in simple yeast systems (159) and Markov Chain Monte-Carlo algorithms (160). The general approach is that a master equation describes the time-evolution of the probability distribution P for times between DNA replication, at which point histone components are distributed to the daughter DNA molecules in conjunction with semi-conservative replication (i.e., half retention of epigenetic marks). The models often assume a two-state or three-state epigenetic marks, where the histone sites are A (Acetylated), U (unmodified), or M (Methylated). At each time-step a random lattice site representing a nucleosome is chosen and one of the biochemical (kinetic) reactions underlying the chemical master equation (CME) are randomly simulated with a probability proportional to their respective rate constants for the methylation or acetylation dynamics (159, 161). Mean-field approximations must be employed to derive equilibria points (i.e., stable epigenetic states/marks). With cooperativity in the epigenetic marks, bistability (presence of two stable equilibria) is observed in the system representing on/off epigenetic states (162). As such, algorithmic information dynamics can be employed in prospective studies to treat these epigenetic switching systems as discrete dynamical systems suited for Ising spin-glasses, cellular automata, or artificial neural networks.

If we assume *s*, the density of marked nucleosomes, is always large and exhibits faster dynamics than the unmodified states, one can take to the limit of large number of nucleosomes the Fokker-Planck Equation (FPE) for the probability P. Also, for the general situation where recruitments of enzymes by active or inactive marks are asymmetric, the steady-state distribution of FPE has at most three fixed points on the bifurcation diagram with a cusp-like catastrophe indicative of critical dynamics (phase-transition) (67). Further, the FPE approximations have been shown to closely match simulations of the CME approach by Gillespie algorithm. For example, the Gillespie algorithm has been used to model polycomb memory systems in simpler model systems (163). Further details of CME and FPE are provided in the respective citations of the studies, and in Uthamacumaran (164).

However, what happens with 3D-conformation dynamics and long-range interactions? Multi-protein complexes and transcriptional marks are involved in the dynamics of epigenetic states, where many types of histone co-modifications are involved. Current models are thus simplified to at most two or three histone modifications. Long-range interactions need to be accounted for in the modelling by integrating Hi-C data with ChIP-Seq tracks. For instance, few groups have previously investigated how long-range looping interactions may be involved in H3K9me3 domain (constitutive heterochromatin) formation by use of Monte Carlo simulations coupling nucleosome turnover with methylation kinetics (157, 165). The stochastic models were able to well-reproduce the chromatin marks seen in experimental ChIP-Seq profiles. Similar studies were shown to reproduce the SETD2-catalyzed H3K36me3 marks from ChIP-Seq tracks, as well (166). Recent studies have shown stochastic chemical kinetics polymer models using Langevin dynamics to better capture histone methylation dynamics than these above-listed methods (167). Regardless of these computational approaches, the lack of 3D-chromatin modelling and a lack of time-points in conjunction with histone spreading dynamics remains the central problem in current computational epigenetics. Predicting critical dynamics in epigenetic remodelling of chromatin states and their resultant cell fate dynamics with limited time-points is a great burden to complex dynamics discovery in (cancer) epigenetic systems.

Various computational models in stochastic dynamics/simulations were discussed for investigating multiscale cancer dynamics, including population dynamics and growth/invasion processes. These coarse-grained stochastic models include molecular kinetics, differential equations-based model systems, Monte Carlo approaches, and Gillespie algorithm. A summary of molecular dynamics in the emerging field of cancer computational epigenetics was also introduced. These simulations can be paired with experimental techniques and other discussed computational toolkits within this paper to better elucidate the behavioral patterns in cancer ecosystems.

## Machine Learning-Driven Causal Inference

While the above-discussed traditional chaos detection methods can verify if the state-space attractor reconstructed from the time-delay embedding of cancer signals is chaotic, they are bound to dimensionality limits. What happens if multiple chaotic attractors coexist in the signaling/expression state-space? Imagine the computational complexity of dissecting the time-series of a network of thousands of genes or proteins within thousands of cells at once. Identifying chaotic attractors in the state-space of such complex networks is an NP-hard problem. While traditional approaches may fail to dissect these complex networks, model-driven and physics-driven artificial intelligence may provide a solution for causal inference. Recurrent Neural Networks (RNNs) are recently emerging as the state-of-the-art machine learning algorithms for the spatiotemporal prediction of chaotic dynamics and attractor reconstruction in complex time-series datasets. They allow the model-free inference of chaotic dynamics from complex datasets. For example, Reservoir Computing (RC), a type of RNN has recently demonstrated applicability in the Lyapunov exponents prediction of spatio-temporally chaotic systems, such as the forecasting of the KS (Kuramoto-Sivashinsky) equation up to a few multiples of the Lyapunov-time (168, 169).

RC computing is a merging line between Liquid-State Machines and Echo-state networks, two types of random recurrent neural networks (RNNs). Liquid State Machines (LSM) are a type of spiking neural networks composed of artificial neurons with threshold activation functions (170). Each neuron is also an accumulating memory cell of random interconnections. On the other hand, ESNs are random, large, fixed recurrent neural networks. Each neuron within this reservoir network produces a nonlinear response signal. ESNs are equivalent to LSMs from a dynamical point of view. Both parallel approaches were recombined to RC computing, the current state-of-the-art machine learning to predict chaos in time-series (171). The RC neural network consists of three distinct layer types: the input layer, the Reservoir, and the Output layer. The Reservoir is a network of nonlinear units forming recurrent loops with random configuration. Only the output layer is optimized by training (adjusting the weights). No Backpropagation is needed for training thus, it is simple and quick (169). Different output layers can be trained for different tasks (i.e., parallel computing).

For a simple reservoir update, consider the input U(n), the states of the reservoir at time X(n), and the output at a given time is y(n). Let W be the connectivity of the nodes of the reservoir. Using some nonlinear function, we can recursively update the network from its data points in the current state. The reservoir update is described by the generic rule: *x*(*n*) = *f*(*Wx*(*n* — 1) + *W^in^ u*(*n*)) and the network’s output computation is given by *y*(*n*) = *W^out^ x*(*n*). Applications of RC include dynamic pattern classification, chaotic time-series generation, and chaos forecasting (prediction). The Lyapunov exponents and chaotic attractors of spatiotemporally chaotic systems can be attained using RC computing. For example, Pathak et al. (169) exploited the RC reservoir dynamics to find the Lyapunov exponents of high dimensional dynamical systems, from which chaotic attractors could be reconstructed. Local and global metrics such as the Kullback-Leibler divergence, cross-validation measures, and mean-square error can assess how accurately the chaotic attractor was mapped by the neural network or how well the Lyapunov exponents were predicted for the chaotic system.

In some ways, one can think of predicting cell fate transcriptional dynamics or signalling dynamics as reminiscent of weather forecasting in tumor ecosystems. Both are multi-dimensional patterning fluid systems with multi-scale dynamics. As such, it may be useful, in general, to adopt AI-driven computational fluid dynamics (CFD) and fluid turbulence modelling approaches in the study of cancer patterning/cybernetics. For instance, Ling et al. (172) used custom Deep Learning architectures with Galilean invariance to approximate the Reynolds’ stress tensor in Navier-Stokes Equations flows in turbulent regimes. More recent examples of this includes machine-learned super-resolution analysis and reconstruction of complex turbulent flow fields (173). Fukami et al. used convolutional neural networks (CNN) and a hybrid down-sampled skip-connection/multi-scale (DSC/MS) model to forecast complex fluid patterns. Another example would be the shallow decoder network by Erichson et al. (174). In such approaches, we can take a few measurements or coarse-grained resolution measurements of the flow dynamics for training the neural networks, and in result forecast/predict its high-resolution flow patterns. However, there are limitations since this is an image-based training method and large, high quality image datasets are required. Furthermore, there are Lyapunov times, windows of predictability, to consider given the 3D-flow evolution of complex structures such as fractal hierarchical patterns and vortices. The reconstruction becomes poorer as we go farther away from the training interpolation region.

More recently, a class of RNNs referred to as liquid neural networks, or liquid time-constant networks, are also emerging as continuous-time neural networks for data-driven time-series forecasting of complex dynamics (i.e., causal inference) (175). These methods remain unexplored in cancer research and modelling/forecasting cancer signaling dynamics. Thereby we should extend these computational models and AI-driven simulation techniques to study cellular patterning systems and chemical turbulence (i.e., intermittent, or spatiotemporally chaotic intracellular flows in morphogens and protein oscillations). There are many other neural networks such as Generative Recurrent Neural Networks with reinforcement learning and other Deep Learning frameworks which can also be trained to detect chaotic attractors in cancer signaling/expression dynamics. The reinforcement learning model is most applicable if the amount of time-resolved data available is very little, wherein the neural network will generate new data which mimics the experimental data for pattern recognition. However, such methods will not be discussed herein.

Various causal inference algorithms and computational systems in the field of machine learning/artificial intelligence are capable of capturing causal patterns/relationships in cancer dynamics. These machine intelligence tools include certain types of neural networks such as reservoir computing, liquid neural networks, and recurrent neural networks. These tools should be exploited in pattern discovery in various cancer processes such as decoding cellular dynamics in gene expression state-space (differentiation dynamics), reconstructing protein signaling networks, and deciphering histone/epigenetic modifications in cancer chromatin-state transitions.

## Algorithmic Complexity

Algorithmic Information Dynamics (AID) is an artificial intelligence platform for causality inference in dynamical systems. AID demonstrates that the algorithmic information of complex networks can be used to steer and reprogram their complex dynamics in phase-space (78, 176). AID provides a set of tools to approximate the Algorithmic (Kolmogorov) complexity of these complex networks and control them *via* merging algorithmic information theory with perturbation analysis in software space. Perturbation analysis can be as simple as the removal of an edge or node from a complex network. A graph network can be represented by a set string or array of binary code. The algorithmic information content of this string/array can then be described by classical measures such as Shannon entropy H(s) or Kolmogorov complexity K(s). The K-complexity, K(s), also known as Kolmogorov or algorithmic complexity quantifies the shortest bits of a string or computer program required to describe a dataset. K-complexity is a robust measure of a network’s complexity vastly unutilized in current approaches to network biology (177).

K(s) may be seen as analogous to Shannon entropy as a measure of complexity (or the lack of complexity, i.e., randomness) (78). However, K(s) is a more robust tool than Shannon entropy to measure the complex dynamics of networks. Unlike our current statistical approaches in inferring complex networks dynamics (such as Shannon’s entropy or correlation metrics), K-complexity provides causal inference of network topology and dynamics. By perturbation analysis using AID tools, one can identify the sub-structures of complex networks driving their information flow and regulating their topology (and in consequence, the cellular states/phenotypes) (78). Although Shannon entropy can quantify the amount of information in a complex system (network), it does not tell us how causally connected they are. Further, entropy provides no insights into the algorithmic content of a graph network. However, the algorithmic information content of a complex network distinguishes a process as a cause or randomness (78). Furthermore, K(s) does not depend on a choice of probability distribution like Shannon entropy does. Therefore, it is more robust than Shannon entropy in measuring the complexity of graph networks, such as cancer plasticity networks. Further, we have shown that Shannon’s information entropy rates is closely matched to lossless compression algorithms in comparison to algorithmic complexity. K(s) is also emerging as a machine intelligence platform to reconstruct attractor landscapes such as the Waddington epigenetic landscape of biological networks and causal discovery in their network state-space dynamics (78, 176).

Formally, the Kolmogorov complexity of a discrete dynamical system s is *K*(*s*) = min{|*p*|: *U*(*p*, *e*) = *s*}, where p is the program that produces s and halts running on an optimal reference universal Turing machine U with input e. K(s) is the length of the shortest description of the generating mechanism (of the network or system). For example, a graph network or system is defined as random (or not having a causal generating program) if the K(s) is about the same length of s itself (in bits). However, *K(s) is semi-uncomputable* and must be approximated using tools from AID.

K(s) can be seen as analogous to a measure of the compressibility or irreducibility of an object such as a string or network, or a dynamical system. Then, K(s) of a network matrix s is the length of the shortest compressed file producing s when decompressing it. Compression algorithms like LZ77, LZ78, Huffman coding, and LZW (Lempel-Ziv-Welch) are some examples of lossless compression algorithms (78). They are closer to Shannon entropy (rate) estimations than the graph complexity since they can detect statistical regularities within the information system. However, currently no compression algorithm can estimate the K(s) of a complex network since they are not sensitive enough for small perturbations. As such, Block Decomposition Method (BDM) (178) can be justified as the most appropriate method to study graph and network complexity perturbation analysis (78) also providing a more sensitive and robust alternative to limitations of entropy-based statistical compression algorithms such as the LZ and LZW family of compression algorithms.

The Coding theorem method (CTM) is based upon, or motivated by, algorithmic probability and is able to provide an estimation to K(s). However, CTM is computationally expensive (i.e., applicable only to short string or small object sizes). Therefore, BDM is available as an extension of CTM. It approximates the K(s) of a dataset, providing local estimates of the algorithmic complexity (78). Let U be an optimal reference universal Turing machine and p be a program that produces s running on U, then, the Solomonoff-Levin algorithmic probability is given by:


$$m\left(s\right)=\sum_{p:U\left(p\right)=s}1/2^{\left|p\right|}<1$$

Then, the shortest program p, K(s), is related to the algorithmic probability by the CTM, which states: *K*(*s*) = —*log*_2_(*m*(*s*)) + *0*(1) (179, 180, 182; 181).

There is also Bennett’s logical depth, a measure based on Kolmogorov complexity, defined as follows:


$$Depth_{s}\left(x\right):=min_{p}\left\{T\left(p\right):l\left(p\right)-K\left(s\right)\leq s,\;\left(U\left(p\right)=x\right)\right\}$$

While the K-complexity measures the length of the minimal program required to generate the string or graph s, the logical depth measures the fastest program(s)/computation time T, i.e., shortest running time length, needed to generate the system (183). This is a very interesting measure because it would capture objects in the chaotic regime and place them as having deep structure even when, to some purposes, are random-looking. They are neither the simplest by their emergent behavior nor algorithmic randomness, but their dynamics require computational time to emerge at a usually small critical interval (9).

The set of graph eigenvalues of the adjacency matrix is called the spectrum of the graph. The Laplacian matrix of a graph is also sometimes referred to as the graph’s spectrum. Eigenvalues of evolving networks can be computed, and one can observe the graph complexity K(G), where G is the graph representing the string s, versus the complexity of the eigenvalues, to obtain information about the amount and kind of information stored in each eigenvalue (178, 184). Further, by assessing the maximum entropy per row of the Laplacian matrix, the eigenvalue which best characterizes the evolving network can be identified. Graph spectral analysis provides a quantitative tool for characterizing attractor dynamics in complex networks. CTM studies dynamical systems in software space, characterizing the effects of perturbations and natural or artificial changes to a system in terms of the changes in the set of the underlying explanatory computational models able to explain the system before and after the intervention (176).

The Block Decomposition Method (BDM) allows to combine the power of statistical information theory and algorithmic complexity hence extending the range of CTM to characterize local but longer-range algorithmic patterns. BDM is defined as: $BDM=\sum_{i-1}^{n}K\left(block_{i}\right)+log_{2}\left(\left|block_{i}\right|\right)$ , where the block size must be specified for the n-number of blocks. When the block sizes are higher, better approximations of the K-complexity are generally obtained. Although all methods listed here are applicable to causal discovery in dynamical systems, BDM is the most useful (and robust) tool in AID so far to study K(G) on all objects, including applications of perturbation analysis to graphs and networks, and as such has the potential to provide a computational framework to quantify the causal structure and complex dynamics of cancer networks (78, 185).

To apply BDM on cancer networks/datasets that operates at a discrete and binary alphabet, one can binarize the underlying adjacency matrices with a moving threshold to obtain a vector of networks and associated BDM values. Then apply the BDM on the vectors and obtain a vector of BDM values to work with. By sampling through all thresholds, the process is immune from the arbitrary choice. The identification of the essential features of complex networks- motifs, cliques, and subgraphs, is an NP-complete problem (78). Therefore, identifying cancer stemness networks is NP-complete, in principle, in traditional approaches unless some *a priori* assumptions are made on the underlying data distribution. However, *algorithmic complexity* and the AID toolbox of measures to approximate K(G) avoid such assumptions of predicted data distributions to fit the complex system and find computable candidate mechanistic models. AID provides a robust platform to identify *causal structures* such as chaotic attractors in cancer networks (78).

Perhaps the most elegant aspect of AID is that it provides a computational description of biological information processing. Unlike our traditional perspective of evolution by natural selection, AID provides a view of adaptive processes as *algorithms* steered by causal information dynamics. In systems science, one often uses the term *cybernetics* to denote the study of information processing (dynamics), regulation, feedback, communication, and control in complex systems. As such, cyberneticians often refer to complex systems as control systems, regulatory systems, or feedback systems. Cells, genes, and proteins, the essential structures of information processing in biological cybernetics, can then be treated as computers, programs and codes forming complex multi-scaled feedback loops and hierarchical structures. AID allows causal discovery in such complex systems. Further, in a recent study, AID measures such as BDM have been demonstrated as powerful tools which could highlight evolutionary paths in biological systems. The algorithmic probability reduces the space of all possible mutations and AID was shown able to detect biological pathways more likely to generate mutations versus those which are more stable (185). These findings demonstrate that evolutionary dynamics may be treated analogous to evolving programs in software space. Therefore, AID measures may provide a robust platform to study the cybernetics (information flow) of driver mutation networks and stemness networks in cancer evolutionary dynamics. These approaches should be extended to the study of cancer systems and CSCs to map their cell fate choices and help identify the minimal set of mutations or driver signals required to confer cancer stemness.

Algorithmic complexity provides a robust screening tool for cancer dynamics under a computational systems framework, whereas cellular processes can be viewed as programs and cells as computers. Network perturbation analysis using algorithmic complexity measures was discussed as a statistically strong method to identify causal biomarkers governing cancer cell fate dynamics.

## Conclusion

In summary, various algorithms for the detection of chaotic attractors in the signaling state-space of cancer networks have been discussed. The basic insights into chaos, fractals, and complex systems have been sowed in the context of cancer dynamics. Although chaos exhibits apparent randomness, it has distinct properties and patterns which distinguish it from stochasticity. More precisely, chaotic systems exhibit emergent structures in their state-space with a (multi)fractal dimension: *strange attractors*. Although mathematical and computational models of cancer dynamics have demonstrated the existence of chaos and strange attractors within cancer cells, their experimental confirmation remains limited. The lack of time-series cancer datasets (largely in part due to technological barriers) and a lack of complexity science in cancer research are fundamental barriers in experimentally detecting complex dynamics in cancer cells. However, there are various emerging ways to acquire time-sequential cancer datasets in single-cell transcriptomics and proteomics, as discussed in the introduction.

**Figure 1:**
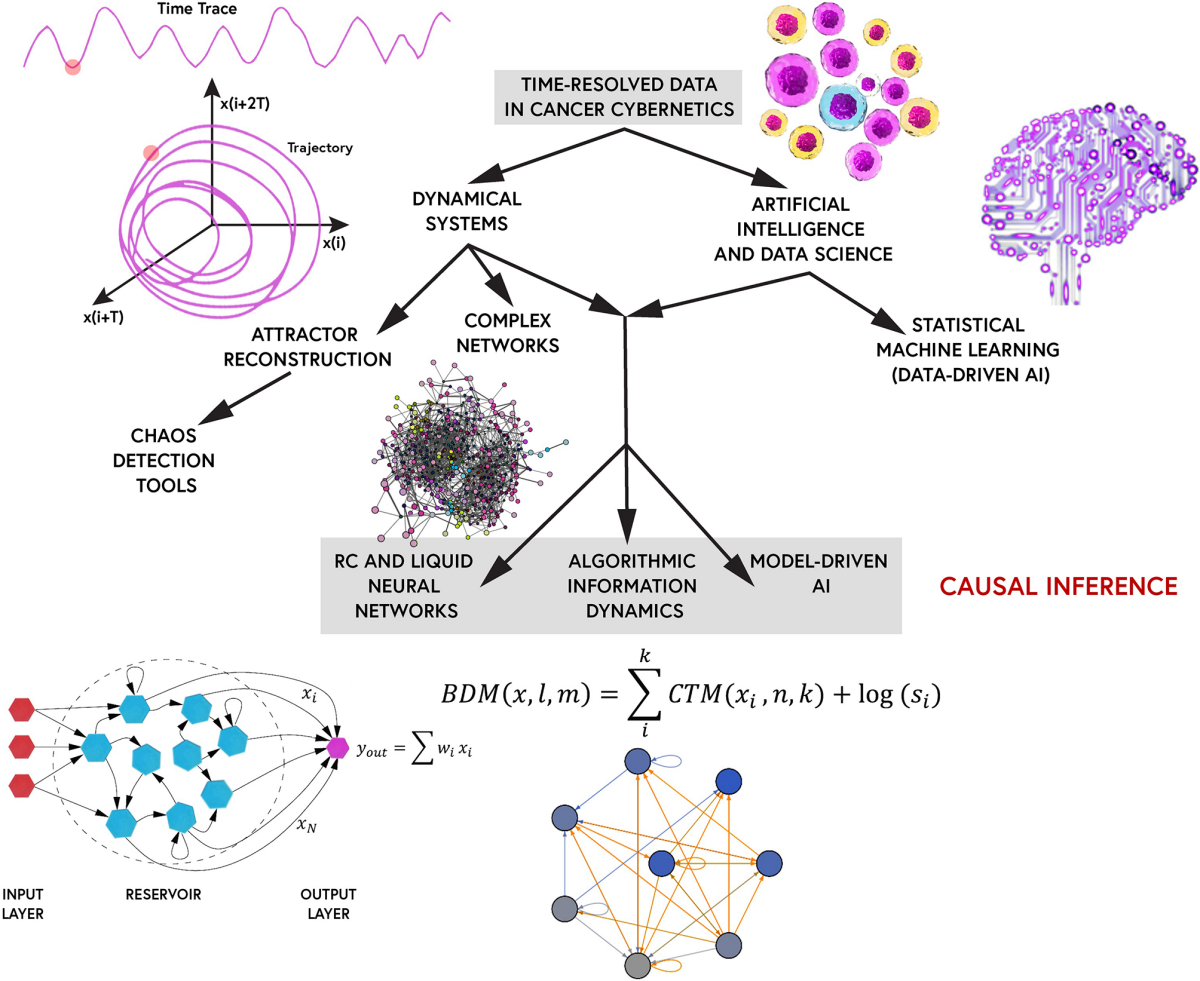
Biological inverse problem. The workflow summarizes a blueprint of causal inference methods and measures discussed in the review for systems oncology. Given time-resolved cancer data (e.g., live-cell imaging of protein flows, time-sequential transcriptomic profiling, etc.), we can employ complex systems tools such as dynamical systems modelling or statistical machine learning algorithms for pattern discovery. Dynamical systems approaches include attractor embedding followed by chaotic behavior detection tools as discussed, or complex networks inference. Chaotic behavior detection tools comprises of many approaches discussed in the paper including attractor embedding, fractal analysis, frequency spectra, and Lyapunov exponents. However, these approaches may have dimensionality limits and hence, AI-driven causal inference algorithms are proposed as promising tools for causal pattern discovery in single-cell time-sequential analyses, which include algorithmic information dynamics (i.e., measuring the algorithmic complexity of complex graph networks via perturbation analysis in software space), recurrent neural networks (e.g., RC networks, liquid neural networks, etc.), and model-driven AI (e.g., turbulence modelling/multiscale computational fluid dynamics).

A blueprint (tree-diagram) of causal inference in time-series cancer datasets is provided in **Figure 1**. The general road map to detecting a chaotic attractor (if it exists) in cancer signaling dynamics is such that first the time-traces of the signal of interest such as gene expression from time-resolved single-cell transcriptomics or protein oscillations from live-cell imaging is acquired (**Figure 1**). Then, it must be embedded *via* time-delay coordinate embedding to be visualized in a three-dimensional space. Following, various discussed algorithms such as fractal dimension, Lyapunov exponents, and entropy measures, can be applied to verify if the embedded pattern is a chaotic attractor(s). There are other chaos detection tools which were not discussed here and could be useful in dissecting biological cybernetics. One good example would be the 0-1 test proposed by Gottwald and Melbourne (186). However, given the dimensionality limits of such traditional techniques like time-delay embedding and topological entropy, the review strongly suggests the exploitation of machine learning algorithms like RC networks and liquid neural networks, and artificial intelligence platforms like algorithmic information dynamics (AID) for causal pattern discovery in cancer systems (**Figure 1**). The techniques outlined in the tree-diagram have widespread applications in systems medicine, including other single-cell multiomics datasets (e.g., protein abundance matrix from CyTOF or histone mass spectrometry, single-cell chromatin modifications matrix from EpiTOF, etc.) (187). **Table 2** in the **Appendix** provides a simplified summary of how the complex systems techniques/tools may apply to different types of datasets and the format of the dataset required for their application.

**Table 2:** Dataset format for complex systems methodologies.

TECHNIQUE/METHOD	TYPE OF DATA	NUMBER OF OBSERVATIONS	LONGITUDINAL OR DISCRETE-TIME	NUMBER OF PARAMETERS
**Takens’s theorem/Convergent Cross Mapping**	Individual	Rich	Both	Minimum 1 dimension for discrete-time and 3 dimensions for longitudinal; and time-delay parameter
**Lyapunov Exponents**	Individual or Mean	Rich	Longitudinal	1-2 parameters (dynamical variable and time)
**Fractal Analysis**	Individual	Scarce or Rich	Both (mainly Discrete)	2 for Box counting technique
**Fast-Fourier Transform**	Individual or Mean	Scarce or Rich	Both	Minimum 2 dimensions (time and variable of interest)
**Entropy**	Individual or Mean	Scarce or Rich	Both	1 or more; *a priori* assumption of statistical distribution for Shannon entropy
**Ising Model/Spin Glass**	Mean	Scarce or Rich	Discrete	1 or more; mean-field approach/*a priori* assumption of statistical distribution
**Cellular Automata (CA)**	Individual	Scarce or Rich	Discrete	1 or more
**Recurrent Neural Networks**	Individual	Rich	Both	Minimum 2 (time and dynamical variable)
**Stochastic Simulations**	Individual or Mean	Scarce	Discrete	Statistical Distributions (*a priori* assumed)
**Differential Equations**	Individual or Mean	Scarce	Longitudinal	2 or more (time and variables); discretization or assumptions are required for analytical solutions
**Block Decomposition Method**	Individual	Scarce or Rich	Discrete	1 or more
**Algorithmic Perturbation Analysis (Graph Network Complexity)**	Individual or Mean	Scarce or Rich	Discrete	1 or more

Chaotic behavior in population dynamics/cellular ecosystems has been predicted as a signature for generating heterogeneity, tumor aggressiveness, metastatic invasion, recurrence/relapse, and therapy resistance (89, 90). Further, intracellular chaos has been suggested as a hallmark of cancer progression and aggressivity herein. Jensen et al. demonstrated the flows of protein densities may form strange attractors within cells (18, 19, 155). In extension of their findings, chaos is suggested as a causal mechanism by which tumor phenotypes can acquire adaptive properties and increase their fitness in harsh fluctuant environments. The detection of chaos within cellular oscillations and protein flows are predicted to be indicators of complex dynamics driving cancer networks. Further, chaotic dynamics in a single transcription factor were shown to orchestrate phenotypic heterogeneity and the enhancement of downstream gene signaling (18, 19). Then, the emergence of intracellular chaotic dynamics at the level of protein flows and gene regulatory networks may allow cancer cells to become highly robust to perturbations, conferring adaptive advantages to dynamic environments (resilience), promote their phenotypic plasticity, and generate aggressive phenotypes with therapy resistance.

**Figure 2:**
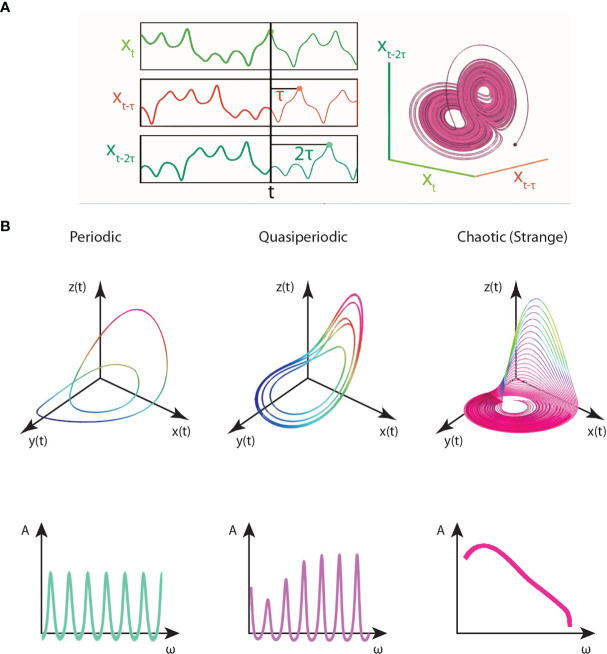
Attractors and oscillations. **(A)** Time-delay Coordinate Embedding. A schematic of attractor reconstruction from a time-series signal of some variable X(t) is shown by time-delay embedding (i.e., Convergent Cross Mapping). τ represent the time-delay. However, for complex large-scale datasets, machine learning algorithms such as reservoir computing (RC) and deep learning architectures are suggested (Image was adapted from 37). **(B)** Three different types of attractors which can self-organize in the signaling/expression state-space of cancer processes are shown: a limit cycle (periodic oscillation), quasi-periodic attractor, and a strange attractor (chaotic). The simplest of attractors, a fixed-point, is not shown herein. Their corresponding frequency spectra are shown below, with the oscillator’s angular frequency as the independent variable and the amplitude of the oscillations as the dependent variable. The oscillation of a limit cycle attractor has a defined amplitude **(A)** and peak in the frequency spectrum at a frequency (ω). A broad frequency spectrum is observed for the strange attractor, which exhibits a fractal-dimension in state-space. However, the frequency/power spectrum can be more complex depending on the system. For instance, complex attractors, such as those observed fluid turbulence, exhibit a broad frequency spectrum with an anomalous power-law scaling (i.e., multifractality) due to intermittency.

Therefore, chaotic, or complex dynamics are not suggested as signatures of cancer pathogenesis herein, for which, as well-established, genetic instabilities and epigenetic abnormalities provide a better causal mechanism. Rather, complex dynamics are suggested as signatures of tumor progression and aggressivity in cancer cell fate dynamics (**Figure 2**). The detection of a chaotic attractor in cancer signaling implies the presence of cellular (disease) state, which is complex, adaptive, and difficult to treat. Hence, perturbation analysis by means of targeted therapies or cellular reprogramming methods can be used to determine which therapy/perturbation results in the loss in instability and complexity of the strange attractor, and thus, help identify effective precision therapies against aggressive cancers like GBM. Network medicine and complex systems analysis provides another tool to help identify these targeted therapies or gene/protein drug targets for the perturbation analysis. If we see the strange attractor (complex dynamics) reduce to more stable attractors such as equilibrium points as indicated by the loss of a fractal dimension of the attractor or non-positive Lyapunov exponents, we may conclude the perturbation is a robust anti-cancer therapy. In principle, such approaches provide a causal framework to not only screen for precision therapies but also control/predict cancer cell fate dynamics and reprogram their phenotypes towards benignity. Further, causal inference methods should be applied to single-cell multiomics and multimodal profiling methods.

**Figure 3:**
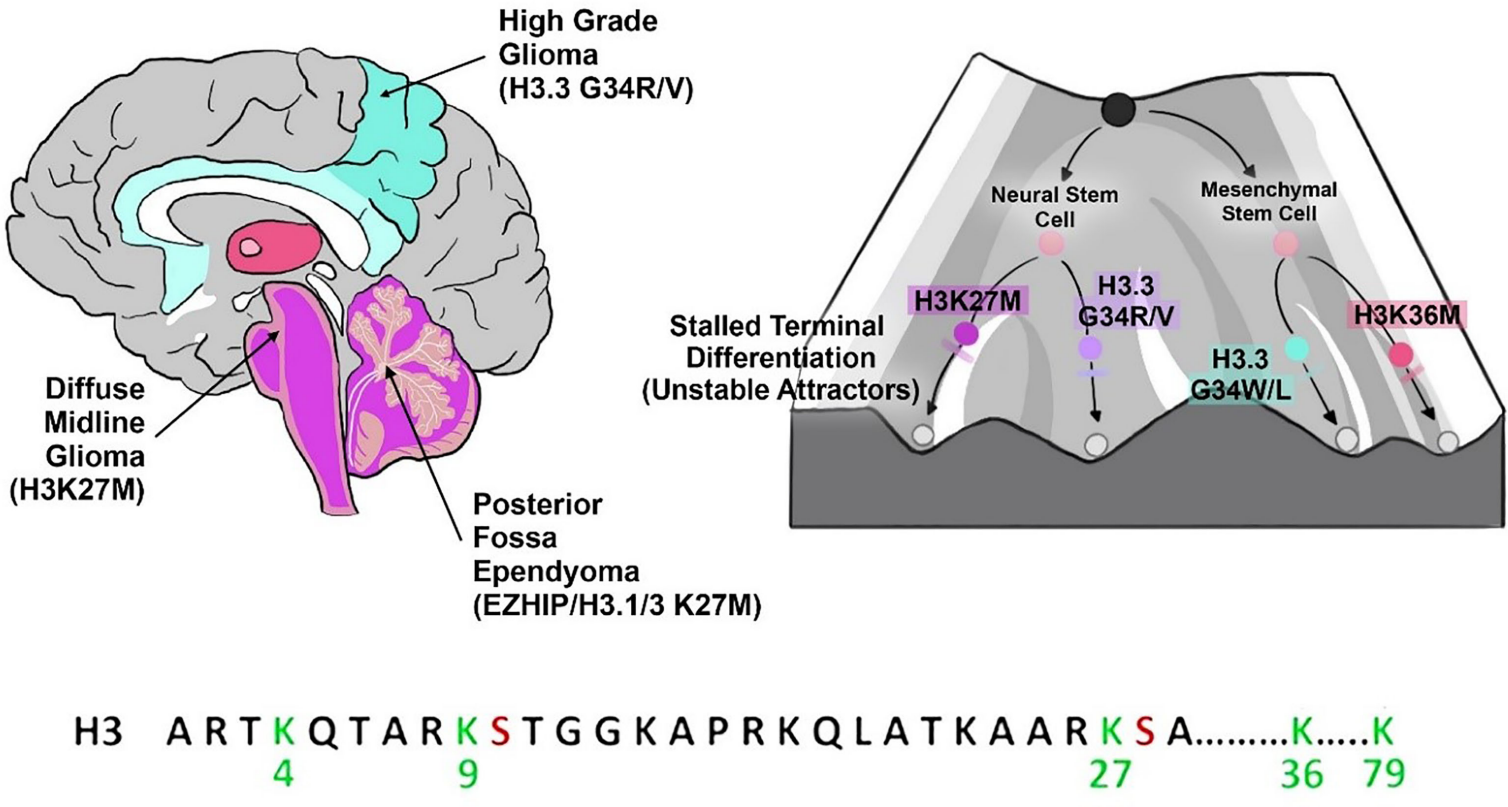
Differentiation dynamics in pediatric glioma systems. On the left, a schematic of the discussed epigenetic variants of pediatric high-grade gliomas (pHGGs) are shown with their corresponding brain regions recapitulating altered neurodevelopmental differentiation circuits. The corresponding Waddington landscape for their stalled differentiation dynamics is shown to the right. The cancer cell fates are shown as stalled attractors on the landscape (gene expression or signalling state-space) resembling stem cell states. Below, a string of the amino acid sequence of the histone tail H3 code is provided with the sites of the recurrent epigenetic mutations in these pHGGs. Some of these epigenetic modifications correspond to active chromatin marks with transcriptional activity while others are repressive marks (inhibited gene expression). The polycomb system is an essential regulator of pHGG differentiation dynamics. The toy-model system provides the biological insights underlying the complex dynamics and mathematical concepts discussed in the paper.

The future of mathematical and computational methods for cancer research holds great promise with the emergence of interdisciplinary fields such as computational oncology and systems medicine. The various tools discussed herein illustrate that they provide quantitative insights into complex cancer processes hindering therapy response and contributing to disease progression/aggressivity, including cancer differentiation dynamics, phenotypic plasticity/stemness, and adaptive heterogeneity. Such complex behavioral patterns allow cancers to develop adaptive traits such as therapy resistance, recurrence/relapse, and metastatic invasion leading to cancer-related deaths. The mathematical and computational tools allow clinician-researchers to dissect the complex networks underlying these behavioral patterns and help elucidate putative therapeutic targets specific to these behaviors. Network medicine and attractor reconstruction allow the control and regulation of patient-derived cancer systems both in silico and in experimental settings, to find novel effective strategies to prevent disease progression or permit cancer cell fate reprogramming. Further, causal inference tools such as algorithmic information dynamics, allow clinician-researchers to decode the causal relationships in driver networks steering the multiscale dynamics of cancer ecosystems, examples of which include transcriptional networks controlling cell fate plasticity and stemness discussed in the paper. Such methods allow treatments tailored towards dynamical responses in cancer therapy since they treat cancers as complex dynamic and adaptive diseases, and hence allow time-dependent progression control-predictability of cancer evolution. These approaches is most beneficial to clinical oncology as it would help pave more effective, cancer network-targeted treatments, precision diagnostics and prognosis with longitudinal screening of patients (e.g., blood-sera biomarkers), and thereby provide extension of cancer patient survival rates. We also predict the complex systems framework of computational/systems oncology may help reprogram cancer (stem) cells to benignity.

## Author Contributions

The article was written by AU under the supervision of HZ. All authors contributed to the article and approved the submitted version.

## Funding

HZ is supported by EPSRC grant no: EP/W004801/1.

## Conflict of Interest

The authors declare that the research was conducted in the absence of any commercial or financial relationships that could be construed as a potential conflict of interest.

## Publisher’s Note

All claims expressed in this article are solely those of the authors and do not necessarily represent those of their affiliated organizations, or those of the publisher, the editors and the reviewers. Any product that may be evaluated in this article, or claim that may be made by its manufacturer, is not guaranteed or endorsed by the publisher.
