# Supplementary material for: A Review of Mathematical and Computational Methods in Cancer Dynamics
Source: Front Oncol. 2022 Jul 25;12:850731. doi: 10.3389/fonc.2022.850731 (PMC9359441; doi:10.3389/fonc.2022.850731)
Supplement: Supplementary file 1 [file DataSheet_1.pdf]

APPENDIX

COMPLEX SYSTEMS: PATTERNS AND BEHAVIORS

The paradigm of complex systems, a multidisciplinary intersection of dynamical systems theory, data science, and various branches of systems science, remains relatively new to most conventional thinkers. As such, we provide here some rich insights into the general features and properties of complex systems. Complexity science is the quantitative study of patterns, processes, and behaviors in complex dynamical systems.

The term *complexity* can also be interpreted as a measure of computational complexity- how difficult it is to solve a problem using the time, space, and algorithmic resources of a computer. Such a computational problem can be classified as P (polynomial-time) vs. NP (nonpolynomial deterministic), or undecidable (Sipser, 1997). In a branch of complexity theory, known as Algorithmic Information Theory, *complexity* is defined by the length of the shortest description (in bits or qubits) of a system. We refer to this information theoretic as the algorithmic complexity or Kolmogorov complexity (Sipser, 1997). The complexity of a state or system is the minimum size of a program that can generate or describe the system (Sipser, 1997). In the study of cancer and cancer signaling, being interested in first succinct, smallest principles, we are interested in the mechanistic models of gene regulatory network or protein-protein interaction network that confers cancer stemness. As such, algorithmic complexity theory is relevant in science in general. In the context of cancer, a cancer stem cell may be interpreted as the minimum size of a molecular network that confers its stemness properties (i.e., self-renewal, differentiation, and therapy resistance). However, cancer ecosystems comprise of interdependent multicellular networks and not the stem cells alone. Further, they exhibit emergent multiscale dynamics requiring the integration of transcriptomics, proteomics, epigenomics, and longitudinal immune monitoring, making such a task (i.e., to define the algorithmic complexity of the system) an intractable, and ultimately, uncomputable problem. This does not mean, however, that algorithmic complexity theory is unable to provide important insights, and that methods should be discarded *a priori*, or that the interaction of information theory with areas such as dynamical systems should be avoided. Signals detected by classical and algorithmic information theory in the form of criticality markers, may be hallmarks of cancer cell fate dynamics and epigenetic memory systems (i.e., phenotypic switching), respectively, and detecting these hallmarks within the cancer networks may provide a solution to overcome the vast search space of this intractable problem.

Throughout the paper, *complexity* will be primarily referring to either *complex systems* (i.e., nonlinear systems exhibiting emergent/collective behavioral patterns) and/or *algorithmic complexity*, a robust measure of non-randomness and computational irreducibility (Sipser, 1997; Wolfram 2002; Zenil et al., 2022). A system is then defined as a *complex system* (non-random) when its algorithmic complexity is not lower than the length of the shortest system (in bits) able to generate it. Complexity thus provides a framework to quantify and characterize complex processes and multiscale behaviors as information dynamics. *Complexity theory* emerged in the 1980s as a discipline of computer science, while the birth of complexity science or complex systems theory was observed around same time. We now know that algorithms can simulate complex systems, and complex systems can simulate algorithms. There is a fundamental principle of correspondence between algorithms and naturally observed complex systems/processes. The key principle of complexity theory is that certain *algorithms are complex systems* (Sipser, 1997; Wolfram, 1988). As such, both (computational) complexity theory and complex systems theory can be merged in time to a single systems framework, which shall be referred to as

complexity science herein (Wolfram, 1988). The central paradigm of complexity science is the realization that any complex system can be represented as *information* dynamics (Zenil, 2022).

The study of chaotic dynamics and its emergent patterns is at the heart of complexity science. In 1963, Edward Lorenz, Ellen Fetter, and Margaret Hamilton pioneered modern chaos theory by studying the unpredictability of hydrodynamic flows in simplified Rayleigh-Benard convection systems as an approximation to weather turbulence forecasting (Lorenz, 1963). There have been earlier pioneers such as Henri Poincare (the father of dynamical systems theory), George D. Birkhoff (ergodic theorem), Mary Cartwright, John E. Littlewood, and Aleksandr Andronov (theory of oscillations and bifurcations), amidst others, who have seen the *fine structures of chaos* emerge before them when studying certain classes of nonlinear differential equations. These *fine structures* are today known as *strange attractors*. The works of Kolmogorov, Sinai, Arnold, Hénon, Smale, Anosov, and others further expanded the study of chaotic dynamics/attractors into the domain of differential geometry (topology) and complex systems such as fluid turbulence. The iconic picture of chaotic behavior and that of a strange attractor is the Lorenz attractor, a multifractal structure observed in the phase-space of their simplified set of differential equations. The Lorenz attractor's intuitive description underlies the famous butterfly effect, i.e., the flap of a butterfly's wings in Brazil may result in the birth of a tornado in Texas.

Most complex systems are high-dimensional *chaotic systems*, or rather, a high-dimensional chaotic system is usually considered a *complex system*. Chaotic systems are inarguably the most complex (difficult to describe) of *complex systems* but not the most complex in the algorithmic randomness sense but the most interesting as they are algorithmic simple, but their unfolding appears complex and sometimes even random (Zenil et al., 2012). The reason being that chaotic systems are unpredictable in the long-term and finding solutions to their underlying equations (if they exists) are analytically intractable, necessitating heuristics or computational methods. For instance, the Lorenz attractor is a computationally solved approximate solution to its differential equations (at certain critical parameters). Notions such as intractability, unpredictability, irregularity (fractality), aperiodicity, irreducibility, and undecidability are inherent to complex systems dynamics. Yet *deterministic chaos* presents another feature of most complex systems: *emergence*. Emergent behaviors define simple, nonlinear interactions at a lower scale spontaneously giving rise to complex hierarchical structures or multi-nested (recursive) patterns in higher scales (Wolfram, 2002). Perhaps this is counter-intuitive as apparently random fluctuations and disorder at one scale of interaction builds up to collective organized behaviors at another. For this reason, chaotic behavior is often referred to as an irregular or strange causal order. Scaling dynamics and the self-organization of these multi-scaled, hierarchical structures are observed in emergent systems (i.e., more is different) (Anderson, 1972). Think of phase-transitions, the flocking of starlings (Vicsek model), the stigmergy of ant colonies, the sudden outbreaks of pandemics, social network dynamics, Jupiter's Great Red Spot, and tumor formation; these are emergent behaviors (Czirok and Vicsek, 2000). Note: Some scholars distinguish emergence from self-organization such that emergence denotes the macroscopically observed collective spatial behaviors in a complex system which cannot be deduced from its many interacting microscopic constituents, while self-organization denotes the emergent patterns/collective behaviors in the temporal dimension. Thus, emergence or synergy denotes the *collective behaviors*/dynamics of nonlinearly interacting parts within a dynamical/feedback system.

To illustrate emergence (or self-organization), individually, ants resemble random walkers with very little intelligence. However, collectively, swarms of ants exhibit large-scale coordinated behaviors and super-intelligence. In the case of the starlings, the flight of individual birds may appear random-like and

disordered. However, nearest neighbor-interactions allow flocks of birds to undergo a phase-transition and exhibit aggregate patterns and behavioral processes resembling the complex multi-scale dynamics of hydrodynamic flows (Vicsek et al., 1995). In the mathematical study of complex systems dynamics, we are interested in the causal patterns to which emergent behavioral patterns are confined to in state space. The self-organization of strange attractors, like the Lorenz attractor, in state-space of the dynamical system is the classic example. Finding these emergent patterns and structures in a complex dynamical system is analytically intractable. Therefore, complexity science advocates the use of computational algorithms and machine intelligence to help find approximate solutions to such complex systems. Furthermore, from an algorithmic perspective, we can distinguish chaotic behavior from randomness by its Kolmogorov complexity (Sipser, 1997) and we shall explore methods to compute estimates of K-complexity in complex dynamical systems.

NONLINEAR DYNAMICS AND CHAOTIC ATTRACTORS

The drastic change of behavior in a nonlinear dynamical system by tuning its critical parameter(s) can demonstrate increasing complexity in attractor dynamics. As we change some set of order parameters, first the dynamical system may exhibit solutions which converge from any initial condition to a static equilibrium value(s) (i.e., a fixed-point attractor). As the values of the bifurcation (order) parameter(s) increase further, the fixed-point attractor undergoes bifurcations and transitions to an oscillation (limit cycle, or periodic attractor). As we further increase the critical value of the bifurcation parameters, the oscillation may eventually make a transition into the chaotic regime (Shaw, 1981). We refer to these transitions of an attractor at some critical point of the bifurcations as *symmetry breaking*. Symmetry breaking defines the phenomenon in which small fluctuations acting on a system beyond a critical threshold abruptly changes (decides) the system's fate, by determining which branch of a bifurcation is taken (Shaw, 1981; Thompson and Stewart, 2002). A stable attractor of a nonlinear system can undergo symmetry-breaking when its order parameter(s) exceed a critical point, above which complex dynamics may emerge. The key insight to understand here is that a fixed-point or a periodic attractor can give birth to a strange attractor as it loses stability (control) and bifurcates from its critical points (Grebogi et al., 1987; Gleick, 2008). Chaotic solutions may be indicators of the most complex types of dynamics where the system exhibits irregularity and long-term unpredictability (Thompson and Stewart, 2002; Gleick, 2008). As such, intuitively, if a cell phenotype or cell signal behaves as a chaotic attractor in state-space, it is difficult to control and predict. However, its cell fate dynamics or signaling patterns are confined to the specific set of states bound to the fractal (strange) attractor. That is, although individual trajectories of the system may exponentially diverge apart, the global orbit/structure of these trajectories are bound to a finite set of state-space (basin of attraction), due to the stretching-folding of phase-space into a fractal architecture (Gleick, 2008; Strogatz, 2015).

One way to visualize the birth of attractors is by observing its bifurcations in phase-space as we tune its order parameters. Another approach is to simply consider the system as a network of coupled nonlinear oscillators. Now consider an external oscillation as a control parameter of this network of coupled oscillators (i.e., cells or genes/proteins). An external oscillatory signal (driving force) can be used to create phase-locking and synchronize the oscillators (i.e., collective dynamics) at some lower critical threshold of the external signal's oscillation frequency (Strogatz and Stewart, 1993; Strogatz, 2004). If the frequency of the external oscillator is strongly coupled to the system, and is tuned at its natural frequency, resonances are observed (increasing amplitudes). However, as we keep increasing the external periodic signal above some critical frequency threshold, the system of coupled oscillators will exhibit Arnold tongues that either causes the entrainment to the external periodicity or results in *aperiodic oscillations* which may further bifurcate towards *chaotic behavior* (Jensen et al., 2012;

Heltberg et al., 2019). Spatiotemporal chaotic behavior can be detected in various routes in a complex system, one approach is to observe *period-doubling bifurcations* in its phase portrait. In the context of oscillators, as discussed, a *broad band frequency spectrum* may be a robust signature of chaos. While periodic oscillators exhibit well defined peaks with amplitudes corresponding to their signal intensity (e.g., concentration of proteins in time, or trajectory of a cell fate transition), a chaotic oscillator will show a broad continuous spectrum. In more complex dynamics, anomalous multifractal scaling may be observed in the system's energy (frequency) spectrum (Jensen et al., 2012; Strogatz, 2015). The power law scaling exponent can be extracted from the slope of the line observed in the log-log plot. However, with many interacting oscillators (cells, molecules, genes/proteins, etc.), it may not be easy to observe such features.

COMPLEX NETWORKS AND MULTISCALE DYNAMICS

Many of the complex emergent behaviors in tumor ecosystems are attributable to a subpopulation of adaptive cells referred to as cancer stem cells (CSCs), found within tumor ecosystems with distinct properties, including self-renewal, therapy resistance, phenotypic plasticity, and the ability to differentiate to multiple heterogeneous phenotypes (Plaks et al., 2015; Xiong et al., 2019). The phenotypic plasticity of CSCs are regulated by their nonlinear interactions with the dynamic tumor microenvironment. Their stemness depend on the signaling networks of their stem cell niche, a complex tumor microenvironment comprised of immune cells, extracellular matrices, blood vessels networks (angiogenesis), and healthy cell networks (Rosen and Jordan, 2009; Plaks et al., 2015). The microenvironmental cues can remodel the three-dimensional chromatin structure within the cellular states by various types of chemical post-translational modifications including histone tail alterations, promoter-enhancer looping, and differential DNA methylation, collectively referred to as *epigenetic modifications*. Epigenetic modifications can be transmitted across cell divisions, and the stability of these epigenetic memory systems govern cellular identity and transcriptional dynamics in tumor ecosystems (Flavahan et al., 2018; Meir et al., 2020). The phenotypic plasticity observed in CSCs and their differentiated heterogeneous phenotypes are governed by epigenetic memory systems. As such, phenotypic plasticity is also referred to as *epigenetic plasticity* (Flavahan et al., 2018). Elucidating the causal mechanisms by which epigenetic switches maintain and promote cancer cell fate dynamics is an active research hotspot for systems medicine.

Due to the advancement of single-cell multi-omics and availability of high throughput epigenetic datasets such as scRNA-Seq, histone mass spectrometry, CyTOF/EpiTOF, scChIP-Seq analyses, WGBS (whole genome bisulfite sequencing/methylome sequencing), scATAC-Seq (single-cell chromatin accessibility profiling), and Hi-C chromatin capture, our recent understanding of single-cell cancer epigenetics have revealed that pediatric cancers are molecularly distinct from their adult counterparts due to a greater epigenetic burden (Schwartzentruber et al., 2012; Wu et al., 2012). Perhaps the strongest evidence to the role of epigenetic modulation of cancer cell fate decisions would be pediatric high-grade glioma (pHGG) epigenetics (Schwartzentruber et al., 2012). Some key insights into the epigenetic and molecular underpinnings of pHGGs are provided herein as a model-system of epigenetic complexity and complex adaptive behaviors in tumor ecosystems, and to help elucidate some of the mathematical models and computational techniques discussed in the later sections. Although in complex systems such as cancers one cannot separate transcriptional dynamics or proteomics signaling from epigenetics, we shall briefly explore glioma epigenetics to acquire some intuition to how adaptive chromatin states give rise to transcriptional heterogeneity and tumor plasticity in cancer ecosystems. Further, as will be discussed, unlike pattern discovery in transcriptional dynamics, computational epigenetics is an emerging sub-domain of systems oncology which necessitates a special class of

algorithms and toolkits pertaining to the study of *critical* phenomena/behaviors. *Criticality* is the state of being poised between regularity and chaos, marked by sudden phase-transition(s) above some threshold control/order parameter of the system (Note: we say regularity instead of order, as it has been traditionally coined, since chaotic behavior is a type of complex, causal/temporal order).

pHGG such as atypical teratoid rhabdoid tumor (ATRT), embryonal tumors, diffuse intrinsic pontine glioma (DIPG), and glioblastoma (GBM) are invasive lethal diseases of the central nervous system (Mackay et al., 2017). They show distinct recurrent oncohistone mutations in the genes encoding histones H3.1, H3.2, and H3.3 (Schwartzentruber et al., 2012, Wu et al., 2012), with the G34R/V (glycine 34 to arginine or valine) and K27M (lysine 27 to methionine) variants indicative of clinically-relevant pathological subgroups. These two mutant oncohistones show altered posttranslational modifications on two key lysine (K) residues of the H3 tail, K27 and K36 due to amino acid substitutions as indicated above. These histone residues regulate complex cellular processes, including developmental genes and embryonic/stem cell differentiation. Further, the emergence of these histone variants also show age and neuroanatomical dependence (Mackay et al., 2017; Deshmukh et al., 2021) (Figure 3).

Roughly 80% of pHGGs of the CNS midline structures (i.e., including the pons, thalamus, and spine) show H3K27M or rarely to isoleucine, substitutions (K27M/I) (Deshmukh et al., 2021). We also see H3.1/2K27M mutations in acute myeloid leukemia (AML), one of the primary hematological malignancies in children. In contrast, G34V/R oncohistones are specific to histone H3.3 mutation (*H3F3A*) which occurs in about 30-50% of cortical pediatric high-grade gliomas and mainly target the temporoparietal cortex (Deshmukh et al., 2021) (Figure 3). There are H3 wild-type gliomas primarily emerging in the fronto-parietal lobes with H3K27M mutations and/or somatic mutations in isocitrate dehydrogenase 1/2 (IDH1/2), thus resulting in excess metabolic enzyme production of 2-hydroxyglutarate (Deshmukh et al., 2021). These epigenetic circuits also reveal how the glioma metabolome forms a bidirectional feedback system with the H3K27 and H3K36 PTM cross-talk, which regulate transcriptional dynamics by recruitment of distinct readers, writers, and erasers (i.e., chromatin remodelling enzymes and proteins). Most chromatin-modifying enzymes require substrates or cofactors that are derived from metabolic intermediates (Kinnaird et al., 2016). This should be self-evident given that our diet/nutrition and drug intake are the primary control mechanisms of epigenetic circuits. The epimetabolic and epiproteomic rewiring in cancer cells provides evolutionary advantages in major cellular decisions, such as proliferation and cell fate differentiation by modulating nuclear transcription, and as such form an interconnected complex adaptive system.

The antagonistic feedback loop between H3K27 and H3K36 methylation dynamics has been revealed as the central regulator of gliomagenesis and glioma progression/cell fate decision-making (Schwartzentruber et al., 2012; Deshmukh et al., 2021). The primary remodelling enzymes mediating these feedback loops are the impaired polycomb repressive complex 2 (PRC2) (H3K27 trimethyltransferase) and SETD2 (H3K36-specific trimethyltransferase) epigenetic memory systems (Schwartzentruber et al., 2012; Huang et al., 2020). There are also enzymes such as NSD1/2/3 mediating the intermediate methylation states in H3K36 di-methylation. A global loss of H3K27me3 due to the inhibition of PRC2 and in conjunction, an aberrant overexpression of H3K36me2 (mediated by NSD1/2) are epigenetic signatures of pHGGs (Schwartzentruber et al., 2012; Harutyunyan et al., 2020). However, how these epigenetic changes affect higher order chromatin structure and oncoprotein signalling or transcriptional dynamics of cancer stemness genes remains unelucidated. The goal of systems oncology should be to employ complex systems theory and tools from artificial intelligence to infer causal patterns in the epigenetic memory systems (e.g., 3D chromatin conformation and histone mark spreading dynamics) in collective behavioral dynamics/processes such as cancer cell fate decision-

making. As will be discussed later, we require computational tools and simulations tailored towards critical dynamics to achieve this complex task. As mentioned, the cellular cybernetics of multicellular adaptive systems like tumors show complex ecosystem dynamics (i.e., collective behaviors). That is, many scales of information dynamics (networks) must be integrated to understand their emergent behaviors and for optimal clinical decision-making in the treatment of these dynamic diseases. Thus, we should integrate the spatiotemporal dynamics at the transcriptomic, metabolic, proteomic, and multi-cellular interactions with these epigenetic datasets to forecast chromatin-state dynamics in complex diseases like cancers

For instance, single-cell molecular profiling and proteogenomics characterization of 218 pediatric brain tumor samples of various histological subtypes including 25 high grade gliomas showed distinct molecular features and signalling patterns in agreement with the transcriptional signatures seen in scRNA-Seq profiles (Petralia et al., 2020). The study also profiled the tumor phosphoproteome, the phosphorylation/kinase activity acting as on/off switches to signalling proteins and observed an upregulation of MEK/ERK/AKT kinases, warranting clinically relevant therapeutic targets. Further, these findings reveal that the information dynamics across different scales of cancer processes, whether it be the transcriptome, epigenome, metabolome, or proteome, to some extent capture interdependent characteristic patterns for causal discovery (Armingol et al., 2021). However, the degree of this correlation depends on the cell type context and microenvironmental complexity. For instance, most proteins expressed in cell communication networks including the cell surface receptors and their ligands are transcribed by the cells, and hence, we can infer protein interaction networks from single-cell transcriptomics (Armingol et al., 2021). We can further dissect the cancer cybernetics at other post-translational modifications at the proteomic and epigenomic levels, including the acetylome, ubiquitylome, methylome, glycoproteome, microbiome, immune interactome, etc. For instance, the role of the gut-immune axis in brain cancers has recently been better elucidated. Further, pHGGs show high amounts of immune cell infiltration, and hijacked immune-inflammatory signals for mediating their tumor microenvironmental dynamics and therapy resistance (immune evasion). There are many other layers of complexity identified in patient-derived liquid biopsies such as the circulating tumor cells, dormant tumor cells (i.e., quiescence), and cancer-mediated exosomes/extracellular vesicles in the maintenance of tumor cybernetics (Li and Nabet, 2019; De Angelis et al., 2019; Park and Nam, 2020).

However, amidst all these multi-scale dynamics in cancer cybernetics, epigenetic datasets require the most attention as its causal mechanisms severely lack understanding. Further, deciphering the epigenetic patterns underlying cancer cell fate dynamics may hold promise to controlling and reprogramming cancer phenotypes and cellular decision-making. Current approaches to cancer therapy including chemotherapy, and radiation therapy are ineffective in the treatment of pHGG. Some recent progress in cancer immunotherapy, and in specific to oncolytic viral therapy are showing potential promises in adult GBM as seen with the PVSRIPO trial (Desjardins et al., 2018). We are also seeing the emergence of epigenetic therapies such as Protein arginine methyltransferase 5 (PRMT5) inhibitors showing promise in disrupting patient-derived GSCs with greater sensitivity on the proneural GBM subtypes (Sachamitr et al., 2021). However, understanding the epigenetic control of GSCs (glioma-derived stem cells) may be the key to preventing glioma progression and reprogramming aggressive cancer cell states to stable phenotypes.

For example, recent in vitro studies have shown the reprogramming of U87MG human glioblastoma cells into terminally differentiated neurons using a small molecule cocktail consisting of forskolin (cAMP agonist), ISX9 (promotes neuronal cell fate differentiation), CHIR99021 (GSK3-inhibitor), I-BET 151 (BET proteins inhibitor), and DAPT (γ-secretase /Notch inhibitor) (Lee et al., 2018). More recently, Gao et al.

(2019) used a combination of Fasudil, Tranilast, and Temozolomide (FTT) cocktail to reprogram patient-derived GBM cells into neuronal-like phenotypes with induced expression of the same neural-specific TFs (i.e., NGN2, ASCL1, etc.) achieved by the above-discussed small-molecule cocktail. Thus, as suggested by these molecular targets and cell fate behavior reprogramming experiments, there are certain signaling pathways and transcription programs such as the GSK3/Wnt pathway and Notch signaling, controlling epigenetic plasticity and differentiation dynamics in glioma systems. However, with a wide set of drug targets such as those used by Lee et al. (2018), it becomes ambiguous which signals are critical for the cell fate reprogramming. As such, we should employ larger drug screens with similar small-molecules or CRISPR screens to identify more robust combinations. Regardless, these studies suggest that perhaps similar lineage-specific transcription factors or chromatin modifiers can force unstable, cancer stem cells or stalled cancer attractors like the pHGGs towards terminally differentiated stable phenotypes (Figure 3). Epigenetic drug targets such as inhibitors of chromatin modifying proteins and histone modification enzymes, such as bromodomain protein inhibitors and CBX inhibitors are also speculated to serve as drugs capable of alleviating the developmental/differentiation blockade in oncohistone variants of pHGGs (Nagaraja et al., 2019). Further, EZH2, the functional enzymatic component of PRC2, is required for glioma stem cell maintenance (Suvà et al., 2009). As mentioned, polycomb dynamics and polycomb memory systems are dysregulated in the oncohistone variants of pHGGs. A deeper insight to the epigenetic and transcriptional circuits regulating these transcriptional programs in relation to polycomb dynamics (and cellular chromatin structure) could help us reprogram and control cancer ecosystems towards benignity.

While the epigenetic profiles are distinct in pediatric gliomas from adults, it remains unelucidated whether similar histone-histone interactions (i.e., combinatorial histone marks), or epigenetic and transcriptional programs also drive the disease in both age groups. For instance, it has been shown that adult GSCs can converge into an epigenetic state reminiscent of paediatric GBM via selective downregulation of H3.3 expression (Gallo et al., 2015; Lan et al., 2017). Hence, similar complex networks may be driving gliomas (cancers) in children and adults, or perhaps the driver complex network's topology undergoes changes from one group to another. These findings warrant further understanding of the epigenetic drivers of glioma stemness/differentiation networks and epigenetic plasticity in complex adaptive tumors. Complex systems approaches will help us quantify cancer cell fate reprogramming and cell fate determinations/decisions as patterns of complex network dynamics and transitions in the stability of attractors on the networks' state-space (landscape). We should repeat the glioma cell fate reprogramming experiments towards forced neuronal lineage commitment discussed above with pediatric glioma samples, including the oncohistone variants, as they are believed to be stalled in their developmental trajectories and more refractive to differentiation/reprogramming (Deshmukh et al., 2021). These studies should be conducted along with healthy controls, to determine whether any side-effects or targets also affect the healthy cells.

Summary: The multiscale dynamics and collective behavioral patterns (attractors) underlying cancer cell fate decisions were introduced herein. The biology-heavy section was intended for non-cancer experts such as physicists, mathematicians, and computational scientists interested in modelling cancer dynamics. Pediatric high-grade gliomas were introduced as a toy model to illustrate the complex multiscale behaviors in tumor cybernetics from epigenetics to proteomics and gene expression patterns. The relationship between 3D-chromatin conformation dynamics, histone/epigenetic modifications, and gene expression profiles in tumor ecosystems was discussed herein. The discussion of key concepts such as epigenetic plasticity, phenotypic switching, and epigenetic memory systems would be pertinent for some of the discussions of mathematical treatments/models in the main paper. These biological insights could help interdisciplinary thinkers investigate causal patterns in multiscale cancer systems/dynamics

using our discussed complex systems toolkits/approaches in the following sections. Further, computational epigenetics is at its infancy in cancer research, and the summarized biological discussions herein could help promote its research program.

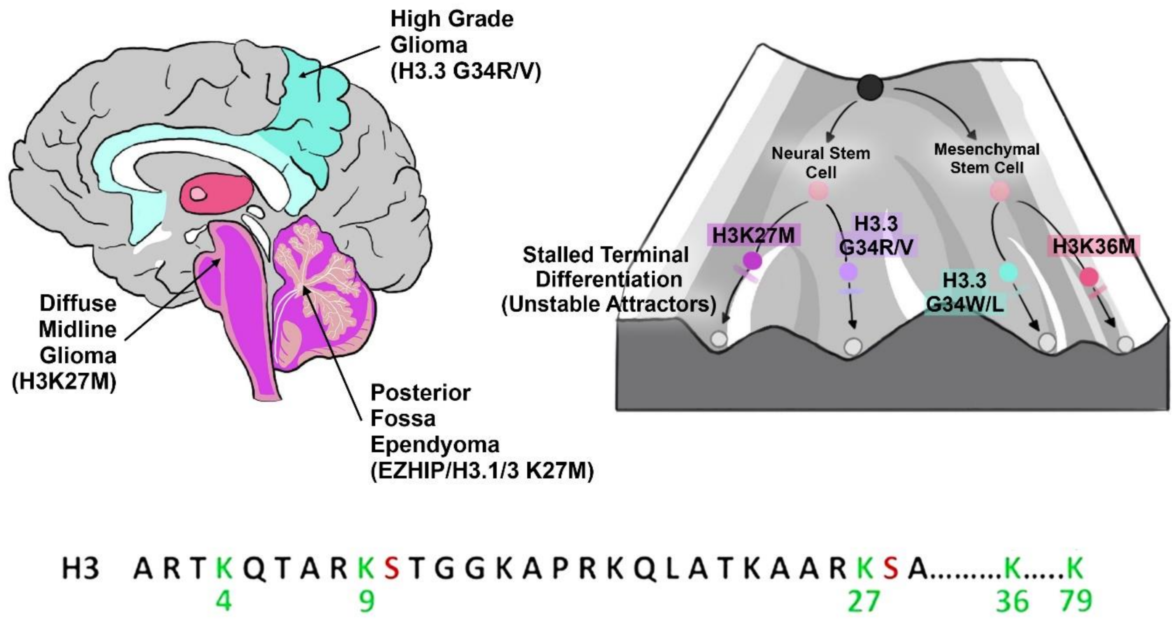

FIGURE 3. DIFFERENTIATION DYNAMICS IN PEDIATRIC GLIOMA SYSTEMS. *On the left, a schematic of the discussed epigenetic variants of pediatric high-grade gliomas (pHGGs) are shown with their corresponding brain regions recapitulating altered neurodevelopmental differentiation circuits. The corresponding Waddington landscape for their stalled differentiation dynamics is shown to the right. The cancer cell fates are shown as stalled attractors on the landscape (gene expression or signalling state-space) resembling stem cell states. Below, a string of the amino acid sequence of the histone tail H3 code is provided with the sites of the recurrent epigenetic mutations in these pHGGs. Some of these epigenetic modifications correspond to active chromatin marks with transcriptional activity while others are repressive marks (inhibited gene expression). The polycomb system is an essential regulator of pHGG differentiation dynamics. The toy-model system provides the biological insights underlying the complex dynamics and mathematical concepts discussed in the paper.*

LYAPUNOV EXPONENTS: INTRACELLULAR PROTEIN FLOWS AND LIVE CELL IMAGING

The Lyapunov exponents can be applied to a wide scale of cancer processes such as the study of protein flows and collective cell (ecological) dynamics. At the scale of protein flows and collective cell migrations, the dynamics behave as a continuum analogous to the hydrodynamic flows of fluids. For instance, when using time-lapse imaging to map cell fate trajectories (as in the case of CSC differentiation mapping), collective cell migration must be considered. Similarly, when imaging the concentrations of fluorescently labelled proteins in a population of CSCs undergoing differentiation (e.g., intracellular morphogenetic flows), the protein patterns within the cells must be treated like a fluid. A vector, velocity field $v(x, t)$ must be defined for such fluid-like systems from the time-lapse imaging data. Various computational tools and software exist for defining the velocity field from imaging data

(e.g., CellProfiler). The Lagrangian flow map F (x_0, t) can then be defined using the velocity field of the intracellular protein flow or collective cellular migration (flocking) patterns, as given by:

$$F(x_0, t) = x_0 + \int_{t_0}^{t} v(F(x_0, \tau) d\tau.$$

The Lagrangian flow maps the initial positions x_0 of the cells or protein flows at time t_0 to their final position at time t (Serra et al., 2020). Using the Lagrangian flow map, we can compute a Lagrangian (continuous) analog of the Lyapunov exponents known as the finite time Lyapunov exponent (FTLE) for the flow trajectories, given by:

$$FTLE(x_0, t) = \frac{1}{T} ln \left\{ \max\left(\frac{|\nabla F(x_0, t)\delta x_0|}{|\delta x_0|}\right) \right\}$$

Where $\nabla F(x_0, t)\delta x_0 = \delta x_t$, T=$t - t_0$, ∇ is the del operator (gradient of F here), and the |·| denotes the Jacobian of the terms inside with respect to initial position. The FTLE measures the maximum trajectory separation rate between the initial position and a neighboring state (protein or cell) starting at $x_0 + \delta x_0$ over time t for continuous dynamical systems (Serra et al., 2020).

FREQUENCY SPECTRA

As discussed in the main text, the interaction strength (coupling) between two oscillators is typically increased by tuning the amplitude of an external oscillator (Heltberg et al., 2019). As the interaction strength between the two oscillators increase, highly complex phenomena may emerge. The range of frequencies of interactions widen, and a period-doubling sequence of bifurcations towards chaotic behavior may occur. In general, a broadband frequency spectrum is observed when oscillators exhibit chaotic dynamics (Figure 1B). An ideal example of this is the Kolmogorov energy spectrum for isotropic, homogeneous fluid turbulence. However, complex turbulent flows, in real-life systems, exhibit intermittency and multifractals, as denoted by an anomalous power law scaling. Since irregular dynamics can be observed in the frequency spectra of complex dynamic systems, specific/general features of the frequency (power) spectral analysis is not as robust as the other discussed chaos detection methods in the review. Hence, we have allocated this space in the appendix to discuss some areas of relevance to which frequency analyses may be useful in dissecting cancer dynamics.

To illustrate a biologically pertinent example, recent findings suggest certain protein flows within cells can exhibit *chemical turbulence*. Chemical turbulence defines the emergence of spatiotemporally chaotic patterns observed in protein-mediated reaction-diffusion systems (Rössler, 1977; Kuramoto, 1984; Halatek and Frey, 2018). Individually, the time-traces of proteins may exhibit Brownian motion. However, collectively, certain protein flows may display fluid-like complex hydrodynamics (Rössler, 1977; Kuramoto, 1984; Halatek and Frey, 2018). A broad band energy spectrum was observed in the chemical turbulence regime of protein fluids within cells reminiscent of the Kolmogorov spectrum of isotropic fluid turbulence (Bohr et al., 1998; Halatek and Frey, 2018). These findings were confirmed in both, simulation and in experiment (Denk et al., 2018; Glock et al., 2019). The emergence of chemical turbulence in cell patterning systems further blur the scaling problem in fluid turbulence.

Fourier analyses have shown the absence of regularity in the oscillations of a cancer signal may be an indicator of chaotic dynamics. For example, an attractor reconstruction via Takens's theorem (time-delay embedding) performed on rat prostate culture oscillations confirmed the presence of chaotic

behavior with a positive Lyapunov exponent. Cellular micromotion was analyzed using an ECIS (Electric cell-substrate impedance sensing), and the corresponding time-series oscillations' Fourier spectra were shown to be broad and flat due to a lack of dominant harmonics. A lack of periodicity and the emergence of a broad frequency spectrum are thereby considered characteristics of chaotic oscillations (Posadas et al., 1998). Even if the system exhibits complex dynamics, as is the case for complex turbulent flows, multifractality may be detected with an anomalous scaling of the broad-band spectrum. Thus, a simple method for chaotic-behavior detection is to subject the time-series signal traces to an FFT (Fast-Fourier Transform) algorithm such as the Cooley-Tukey algorithm. FFT algorithms are built into MATLAB as a 'fft' command, or virtually found in any programming language or graphical analyses software (See Appendix). The method also allows the extraction of power spectral density analysis, which may be more useful for classifying more than one system exhibiting complex dynamics (i.e., spot characteristic patterns distinguishing the two systems). The efficiency of this method also relies on how much the signal is sampled. The resolution of time sampling, and noise filtering must be considered in the Fourier transform analysis of time-series.

Summary: Patterns of frequency spectra in chaotic systems were briefly introduced in the context of cancer signaling dynamics and cellular oscillations. Although a broad-band frequency spectrum may be a signature of chaotic systems, most complex systems exhibit irregular features in their spectra.

CRITICAL DYNAMICS AND NETWORKS

Criticality is the governing principle underlying *phase transitions*. For instance, think of the tangible properties (i.e., density, pressure, etc.) of a liquid in comparison to a gas or solid of the same homogeneous substance. These are first-order phase-transitions. A steam of water molecules and an ice cube are two distinct *phases* of the same system. The critical points are the points at which the distinct phases are at equilibrium. There are large density *fluctuations* near the critical point of a system (Sethna, 2006). Under a microscope, there would be patches of liquid embedded into patches of gas molecules when the critical temperature is approached. A practically insightful model to visualize phase transitions and quantify their dynamics are spin glasses such as the Ising model, consisting of a set of coupled, interacting magnetic dipole spins on a lattice, where each spin configuration corresponds to some energy. Below or above a *critical temperature*, symmetry-breaking occurs resulting in a phase-transition from a random spin orientation to alignment (order). However, the optimization (finding the ground state energy) of a 2D Ising model is an NP-hard problem. Think of biological complex systems, where one deals with an order of magnitude in $\gg 10^{23}$ molecules, and hence, the Ising model becomes combinatorially complex (Mezard and Montanari, 2009). However, an effective mean-field theory can be derived for energies below a certain cut-off of the system (critical point) using renormalization groups (Mezard and Montanari, 2009).

Power laws are the signatures of criticality. They correspond to straight lines in log-log plots and are characterized by the generic distribution $n(s) = ks^{-\alpha}$, where k is some constant, n(s) is the size distribution, s is the size (variable of interest) and α is the scaling exponent. Debated evidence to the universality of critical dynamics include earthquakes, solar flares, stock market crashes, social networks, financial/economic networks (i.e., Pareto's principle), pandemics, the internet, etc. as they obey power-law distributions and scale-free networks (Bossomaier and Green, 2000). For example, consider the Pareto's principle where the rich get richer in a power-law behavior. This occurs due to the interconnectivity of socioeconomic networks (i.e., hubs formation). One of the mechanisms for criticality is known as *preferential attachment*, where whether you grow or not in time depends on how big/connected you already are. Another mechanism for critical dynamics is *self-organized criticality*

(SOC). SOC defines the tendency of a complex system, often a slowly driven nonequilibrium system, to spontaneously gravitate towards critical phase-transition points for a wide variety of initial conditions (Bossomaier and Green, 2000). Scale-free biological networks tend to exhibit hub markers or hub genes, which are putative therapeutic targets and control switches of cellular dynamics/cybernetics.

To obtain an intuitive picture of SOC, we can consider the Per Bak's sandpile model. Bak et al. (1987) demonstrated using a cellular automaton that each time a grain of sand is added to a pile of sand on a checkerboard/lattice, if the number of sand grains on a square exceeds a critical threshold (tipping point), the pile topples forming an avalanche (in a multidimensional model) and gives sand to its neighbors. The distribution of these avalanche sizes and their lifetimes form *power laws*. As we approach larger and larger numbers of sand grains, in the scale of a million or more, Tibetan mandala-like fractal patterns emerge. Recall that fractals are essentially power law scaling, as well. Therefore, power laws are measures of the tendency of complex systems to *transition to chaos*. A clear example would be the transition of a fluid flow from a laminar phase to a turbulent state. The phase transition to turbulence demonstrates an abrupt change of behavior as some order parameter (the Reynolds number) reaches a critical value and undergoes symmetry-breaking bifurcations. In Kolmogorov's model of isotropic fluid turbulence, the energy spectrum exhibits a power-law decay in the inertial subrange with a critical exponent of -5/3, given by: $E(k) = C\varepsilon^{2/3}k^{-5/3}$, where ε is the energy flux, k is the wavenumber, E is the energy of the eddies and vortices, and C is some constant (Ruelle, 1995). The turbulent eddies and vortices breakdown into a fractal hierarchy of smaller eddies and vortices within this range (i.e., the Richardson-Kolmogorov energy cascade) (Ruelle, 1995).

Critical dynamics can occur at many scales of cancer cybernetics, from cellular states driven by gene regulatory networks (GRNs) to chromatin states driven by 3D-chromatin organization and epigenetic regulations. In fact, power law scaling, the signature of critical dynamics, suggests scale-invariant patterns and behaviors. Critical dynamics have been observed in complex signaling networks. Most biological networks exhibit non-trivial topologies including critical or scale-free networks, wherein the network degree distribution obeys power law behaviors, where only a few nodes (hub nodes) have numerous edges while the majority have fewer and fewer links (Barabasi and Oltvai, 2004). These topologies are robust to failure when random nodes are added or perturbed, but vulnerable to the failure of the hubs. The network connectivity in many cancer driver gene networks and protein-protein interaction networks (PPIs) are suggested of power law relationships in their degree distribution (Barabasi and Oltvai, 2004). Let P(k) give the probability that a selected node has exactly k links. The degree distribution is obtained by counting the number of nodes N(k) with k-links and dividing by the total number of nodes N., The degree distribution of scale-free networks obeys a power law $P(k){\sim}k^{-\gamma}$, where γ is the degree exponent (Barabasi and Oltvai, 2004). Smaller the γ, the more important the role of the hubs in the network.

In general, universal properties of scale-free networks exist if $\gamma < 3$, where the dispersion of the distribution $\sigma = \langle k^2 \rangle - \langle k \rangle^2$ increases with the number of nodes, and higher the degree of robustness against accidental node failures or random perturbations (Newman, 2003; Barabasi and Oltvai, 2004). There are many other biological network topologies exhibiting power-law degree distributions, such as the Watts-Strogatz small-world networks and the Barabasi-Albert random networks, not discussed herein (Barabasi and Oltvai, 2004). For instance, the Watts-Strogatz small-world networks are observed in metabolic networks and social networks (Barabasi and Oltvai, 2004). Further, there are network centrality measures (e.g., betweenness, eigenvector, closeness, hub-score, etc.) and modularity (community structure detection) available as measures to explore the regulators of information flow and multi-nestedness of complex dynamic networks, respectively. Network centrality measures such as

betweenness and eigenvector centrality can be used to identify the regions where these hubs occur in scale-free or complex networks (Freeman, 1979; Landherr et al., 2010).

Critical dynamics have been demonstrated in epigenetic processes, as well. As such, the study of critical systems may be most relevant to the emerging field of computational epigenetics discussed in the main text. Criticality is seen in 3D- chromatin spatial organization which is captured by Hi-C data contact maps and super-resolution imaging (Lieberman-Aiden et al., 2009; Mirny, 2011; Boettiger et al., 2016). The promoter-enhancer regions in chromatin Hi-C contact maps were fitted by power law decay models (Lieberman-Aiden et al., 2009, Mirny 2011, Ron et al., 2017). That is, their contact probability as a function of genomic distance forms a power law decay. We see the emergence of hierarchically organized structures in these enhancer loops called topologically associating domains (TADs) revealing a scale-free pattern (Ron et al., 2017). The domains' boundaries were shown to act as regulatory insulators which control phenotypic transcriptional programs by preventing the expression of outside of the enhancer domain. The TAD borders are also enriched genes with high transcriptional dynamics, as well as cohesin and CTCF binding sites, thus supporting the loop-extrusion model (Fudenberg et al., 2016).

There is an open question of whether histone modifications are also poised at criticality? Computational models reveal that a cusp-like phase transition (catastrophe), as seen in EMT dynamics modelling, can be seen in the simulated bifurcation diagrams of chromatin state dynamics (Jost, 2014). These models capture how the recruitment dynamics of epigenetic modification enzymes allow the formation of long-lived (stable) epigenetic memory systems and the stability of epigenetic/cellular identity. Further, they show that critical dynamics with cusp-like catastrophes (tipping points) in the system's bifurcation diagrams, analogous to those observed in Ising spin models, defines the epigenetic plasticity of cell fates (Jost, 2014). Epigenetic switching during phenotypic/developmental transitions and patterns of cell fate behaviors (network dynamics), as seen in cancer ecosystems, may thus be explained by criticality and require criticality detection tools. We must further understand how the bistability in epigenetic/chromatin states transitions to the multistability and metastability seen in phenotypic landscapes (transcriptional dynamics). It is proposed herein that the mechanism underlying this stability bifurcations is a *criticality to chaos transition* from epigenetic/chromatin states to transcriptional dynamics, in tumor ecosystems. Dynamical systems theory and complex systems tools are thus required to elucidate how the critical point bifurcations evolve to the complex dynamics seen in disease-state epigenetic landscapes. Furthermore, phase-transition is not to be confused with the liquid-liquid phase separation seen in certain nuclear transcriptional condensates and protein complexes/transcription factors (Zhang et al., 2019). The liquid-liquid phase separation of intrinsically disordered proteins (IDPs) are emerging as therapeutic targets for reprogramming oncogenic cell fates (Shin et al., 2018; Klein et al., 2020).

CHAOTIC OSCILLATIONS

In the main text, the works by Jensen et al. were discussed as examples of pairing computational simulations with empirical cellular data to investigate intracellular chaotic oscillations/dynamics. Some fine details of these studies and their prospective suggestions are put forth in this appendix subsection. To find the regions of parameter space that exhibit chaotic flows, Jensen et al. first computed the standard deviation in the NF-kB amplitudes from each time series and found the parameter points at which this grew discontinuously, as the external TNF amplitude was increased. Within these regions, they further tested for chaos by computing the divergence of trajectories that started near the initial points, using deterministic simulations. Parameter regions where such trajectories diverged

exponentially were defined as chaotic regions. All deterministic simulations were performed by numerically integrating a set of dynamical equations characterizing the TF-negative feedback loop using the Runge–Kutta fourth-order method, and for optimisation, some of the equations were simulated using Euler integration (Heltberg et al., 2019). All stochastic simulations of NF-kB dynamics were simulated by the Gillespie algorithm. For noise in the external TNF oscillations, Langevin simulations of the different oscillations were used.

These simulations concluded that the flows of protein densities may form strange attractors within cells. Chaotic dynamics in cellular signaling were suggested to allow adaptive heterogeneity to emerge within cell systems. The major findings of these simulations on chaotic dynamics in cellular protein oscillations can be summarized as follows:

- Chaotic dynamics in the oscillation of a single TF NF-kB regulates downstream genes causing their cascading expressions and protein production. The TF plays a vital role in immune homeostasis and cancer stem cell niche dynamics.
- Chaotic dynamics upregulates low affinity genes.
- Chaos increases the efficiency in protein complex formation. The formation of protein complexes are essential for transcription and gene activity regulation.
- Chaos generates dynamical heterogeneous populations of cells and increases their survival in harsh, fluctuant environments. This provides adaptive selection of chaotic cell states to cytotoxic drugs such as chemotherapies.

As suggested by these findings, chaotic dynamics can be used by cancer cells to selectively adapt to their harsh environmental conditions and upregulate certain proteins or specific protein complexes needed for tumor growth and cancer stemness/plasticity. To verify these computational models, these findings should be experimentally validated within cancer cells, especially given that these protein signals are critical driver signals in cancer stemness networks and phenotypic transition/EMT programs. Furthermore, we know of many cancer subset-specific molecular driver network patterns. For instance, Suva et al. identified a core set of four essential transcription factors governing GBM stemness and GSC decision-making (Suvà et al., 2014). The simulations and computational models performed on the NF-kB model can be adapted to these GSC transcription factor networks.

LIMITATIONS AND PROSPECTS

There are many technological and financial barriers to such time-resolved cancer data acquisition. However, what we propose is the time-series measurements of a single patient-derived biopsy's cell culture by means of multi-modal profiling techniques. For certain techniques like live-cell imaging of protein flows or time-lapse imaging of in vitro cellular differentiation, this could be relatively feasible. However, for techniques such as time-resolved transcriptomic profiling, traditionally it may have been more difficult as the preparation of a new cell culture (even if from the same patient tumor) at a different time point may exhibit different set of dynamics. Regardless, we could always perform in vitro differentiation experiments with cancer cell cultures or organoids subjected to different growth conditions (e.g., in stem cell media versus differentiation media) or drug perturbations, and acquire their single-cell measurements (transcriptomics, proteomics, single-cell epigenetics/chromatin modifications profiling, cell patterning imaging flows, etc.) as distinct time-points. A good example would be the time-sequential bulk RNA-Seq data from murine models used by Rockne et al. (2020) to forecast acute myeloid leukemia (AML) development/progression. Since this was a leukemia model, longitudinal blood samples were easily acquirable from the mice to perform the RNA-Seq. It should be noted that analyzing

changes in tumor dynamics in combination with drug perturbation analysis (or similar genetic/proteomic modification) comprises of the regulation of multifactorial processes. There are various drug effects parameters such as drug toxicity, specificity, cell type-dependent dosage, distribution (pharmacokinetics/dynamics), etc. which should be considered. For instance, the agonist of a certain receptor or gene upregulation/downregulation could affect many other proteins signalling or gene expression dynamics. However, with well-defined controls and the discussed complex systems tools (attractor reconstruction and network analysis), the transition dynamics from control states to drug perturbed states can be mapped, and if causal patterns of network or state-transition dynamics exist, in principle, the combination of discussed tools can trace/decode the causal mechanism.

However, in the case of difficult tumors such as high-grade gliomas and other brain tumors, transcriptomic screening of biopsies at different time points are not feasible with human patients. As such, we could either perform time-sequential measurements on xenograft mice models with human patient-derived tumor biopsies, or alternatively, we can perform in vitro experiments with differing growth media conditions or pharmacological perturbations and/or cell passages to mark the time points, as mentioned. Surgeries can be performed on the xenografted mice tumors over the time-span of days if not months to analyze time-course expression patterns. A recent example of mice tumor models-based time-resolved RNA-Sequencing studies includes the study of BRCA1-associated mammary tumorigenesis dynamics by Bach et al. (2021).

Thus, recent advances are bypassing these technological limitations in scientific causal discovery. There are emerging droplet microfluidics-based techniques such as single-cell metabolically labeled new RNA tagging sequencing (scNT-seq), proposed to offer massively parallel time-resolved single-cell analyses within the same cell samples (Qiu et al., 2020). Another emerging technique is the combined use of fluorescent-reporters and live-cell imaging to acquire time-series profiles of selected genes (or protein) expression dynamics with sampling time rates in the hours scales. For instance, Krenning et al. (2021) developed a method combining live-cell microscopy and FACS-analysis of the FUCCI fluorescent reporter system with scRNA-Seq to acquire high-resolution, time-resolved transcriptomic profiles of mitotic cells at the M-G1 phase-transition. Similar methods can be exploited to acquire high-resolution time-resolved transcriptomics for other types of cellular processes and phenotypic transitions observed in cancer cell fate dynamics. These droplet microfluidics-based techniques and fluorescent-reporters based live-cell imaging techniques should be exploited in the time-resolved differentiation mapping of cell fate trajectories and their time-resolved single-cell multiomics from tumor biopsies/cancer stem cells in the conditions described above (Qiu et al., 2020).

To conclude, chaos/complex dynamics are suggested as hallmarks of cancer stemness and cancer progression and should be screened in the gene expression/protein signaling state-space of cancer stemness networks. Moreover, *criticality* has been proposed as a causal mechanism driving cancer epigenetics and chromatin state organization. The limits of current simulations approaches have also been presented in forecasting the dynamics of epigenetic memory systems in cancer cell fate decisions and differentiation dynamics. Further, although the oncohistone profiles of pediatric gliomas are molecularly distinct from those of adult patients, whether the same driver pathways are steering their cell fate decisions remains an open query. As opposed to our current dogmatic approaches based on snapshot statistical patterns and correlations, the detection of underlying causal attractors would pave a novel research programme, with targeted dynamical therapies in the frontier of systems oncology and precision medicine. Elucidating the complex networks and attractor dynamics governing the transition from criticality (in epigenetic regulation) to chaos (in transcriptional/cell fate dynamics) in CSCs may pave the reprogramming of cancer cell fates to benignity.

RELATIONSHIP BETWEEN DISCUSSED TOOLS AND METHODS

The various chaos detection tools and methods for causal pattern discovery are interrelated. For instance, entropy is related to fractal dimension as a measure of chaoticity. Higher topological entropy implies higher uncertainty and information flow (Note: thermodynamics uses the term disorder. However, in complexity and statistical physics, the term irregularity or uncertainty/unpredictability is preferred to denote high entropy since chaos is a form of causally complex order). Similarly, a higher fractal index implies higher statistical self-similarity but also higher irregularity. Therefore, entropy and the fractal index are both measures of irregularity in complex dynamics. Topological entropy is also related to the Lyapunov exponents of a chaotic system. As discussed, a positive Lyapunov exponent is a signature of chaotic/complex dynamics. The Margulis-Ruelle inequality and Pesin's entropy formula show that the entropy of a measure that is invariant under a dynamical system is obtained by the total asymptotic expansion rate of the system. The exponential expansion rate of the phase-space dynamics of a chaotic system (i.e., the stretching-folding of state-space) is measured by the Lyapunov exponent(s). The topological entropy is bound by the sum (integral) of positive Lyapunov exponents for a chaotic system (assuming ergodicity) (Hasselblatt and Pesin, 2008). The Takens' embedding theorem or CCM are not directly related to these chaos discovery tools, rather it is the fundamental step taken in the study of dynamical systems to reconstruct the underlying attractor (state-space), given a time-series signal (i.e., attractor reconstruction) (Sauer, 2006). The time-embedding of the signal is then subjected to these various chaos detection tools such as fractal index, Lyapunov exponents, entropy, etc. or causal inference methods such as algorithmic complexity measures or machine learning algorithms for pattern discovery. Takens' theorem and CCM have dimensionality limits, hence, machine learning algorithms, such as the neural networks or physics-model driven methods discussed in the main text are emerging as attractor reconstruction methods for complex dynamical systems.

Algorithmic complexity, K(G) for a graph network G, remains the most robust causal inference tool in the study of computational/complex systems. In algorithmic information dynamics, entropy rate is analogous to lossless compression algorithms, while algorithmic complexity estimates by the Block Decomposition Method (BDM) provide a more robust, and accurate quantification of complex dynamics (Zenil et al., 2019). Further, we discussed various machine learning tools for causal pattern discovery such as liquid neural networks and reservoir computing. It should be noted that these tools are tailored towards finding statistical associations/patterns and not necessarily causal inference. As such, algorithmic complexity remains the central algorithm discussed in the paper optimized for causal inference in complex systems/networks. There are other tools such as the methods developed by Judea Pearl (e.g., Bayesian networks) and certain types of deep learning architectures that could fall within causal inference methods not discussed in the review (Pearl, 2009).

TABLE 1: GLOSSARY

METHOD	DESCRIPTION
Takens' theorem	A technique for embedding the time-series signal in state-space using a time-delay in one of its coordinates. Convergent Cross Mapping is an embedding algorithm implementing Takens' theorem, applicable on complex networks. The technique has dimensionality limits and hence, should only be limited to a few signals with predicted chaotic dynamics.
Denoising Algorithms	Any algorithm intended for noise reduction. Can range from filtering and preprocessing tools (interpolation, smoothening, etc.) to wavelet-analysis methods. Imputation algorithms are emerging as popular candidates. Not discussed in detail since it consists of a wide range of algorithms, the applicability of which depends on the type of dataset and system of interest.
Lyapunov Exponents	Measures how fast two initially close points on a chaotic trajectory exponentially diverge apart in time. Positive Lyapunov exponent(s) are characteristic signatures of chaos.
Fractal Dimension	Fractals are the geometry of chaos. A fractal is a geometric pattern exhibiting statistical self-similarity (i.e., power law scaling) across many length and time scales with a fractional (non-integer) dimension. It is used as a measure of irregularity, roughness, and complexity. Some algorithms to estimate the Fractal Dimension include the Box-counting method, Fourier analysis-based approaches, and the sandbox method.
Multifractal Analysis	If more than one fractal dimension is required to describe the complexity of the system, multifractal analysis is required. These approaches are most applicable for time-series analysis. The local Holder exponents and the Hurst index are pertinent measures. Wavelet Transform-based methods remain the most popular tools for identifying these multifractal statistics.

Fast-Fourier Transform (FFT)	The frequency and power spectra of time-series signals can be acquired using FFT. The FFT algorithm decomposes a time-series into its constituent frequencies. Chaotic systems generally exhibit a broad frequency spectrum.
Criticality	Power laws are indicators of critical dynamics, a state of hierarchical self-organization poised between regularity and chaos. When certain complex systems surpass their critical point, they gravitate towards chaotic dynamics. The Ising model is discussed as a powerful tool to model criticality in cancer gene expression and patterns of network dynamics.
Entropy	Maximal entropy and a positive entropy rate are observed in dynamical systems exhibiting increased chaotic flows in phase-space. They could be indicators of phase-transitions to chaotic dynamics and/or the birth of complex attractors. However, entropy is not a robust measure of network (graph) complexity and may fail to distinguish randomness from chaoticity.
Computational Modelling and Simulations	The pairing of simulations/computational modelling with data science is the central principle of complexity science. Herein stochastic simulations such as the Monte Carlo methods and Gillespie algorithm were discussed for simulating chemical kinetics and molecular dynamics.
Recurrent Neural Networks (RNN)	Reservoir Computing (RC) networks and liquid neural networks are the state-of-the-art Deep Learning Networks for time-series forecasting and spatiotemporal prediction of chaotic dynamics from complex, multidimensional datasets.
Algorithmic Complexity	Also known as the Kolmogorov complexity (K(s)), is a measure of the length of the shortest description of a dataset (e.g., a string, an array, a network, or dynamical system) or the shortest program needed to generate the dataset. Various algorithms exist for estimating the K-complexity. CTM and BDM (Block Decomposition Method) are alternatives to statistical compression algorithms and are native to n-dimensional complexity.

TABLE 2: DATASET FORMAT FOR COMPLEX SYSTEMS METHODOLOGIES

TECHNIQUE/METHOD	TYPE OF DATA	NUMBER OF OBSERVATIONS	LONGITUDINAL OR DISCRETE-TIME	NUMBER OF PARAMETERS
**Takens's theorem/Convergent Cross Mapping**	Individual	Rich	Both	Minimum 1 dimension for discrete-time and 3 dimensions for longitudinal; and time-delay parameter
**Lyapunov Exponents**	Individual or Mean	Rich	Longitudinal	1-2 parameters (dynamical variable and time)
**Fractal Analysis**	Individual	Scarce or Rich	Both (mainly Discrete)	2 for Box counting technique
**Fast-Fourier Transform**	Individual or Mean	Scarce or Rich	Both	Minimum 2 dimensions (time and variable of interest)
**Entropy**	Individual or Mean	Scarce or Rich	Both	1 or more; a priori assumption of statistical distribution for Shannon entropy
**Ising Model/Spin Glass**	Mean	Scarce or Rich	Discrete	1 or more; mean-field approach/a priori assumption of statistical distribution
**Cellular Automata (CA)**	Individual	Scarce or Rich	Discrete	1 or more

**Recurrent Neural Networks**	Individual	Rich	Both	Minimum 2 (time and dynamical variable)
**Stochastic Simulations**	Individual or Mean	Scarce	Discrete	Statistical Distributions (a priori assumed)
**Differential Equations**	Individual or Mean	Scarce	Longitudinal	2 or more (time and variables); discretization or assumptions are required for analytical solutions
**Block Decomposition Method**	Individual	Scarce or Rich	Discrete	1 or more
**Algorithmic Perturbation Analysis (Graph Network Complexity)**	Individual or Mean	Scarce or Rich	Discrete	1 or more

The table summarizes the type of data (individual counts or mean), the number of observations (rich or scarce), continuous or discrete-time, and the parameter estimations required for the major complex systems tools and chaos detection measures discussed in the review. The table is a generalization as variations may apply to context-dependence and different systems or processes of interest. In general, these tools can be applied for the attractor reconstruction and network analysis of single-cell cancer multiomics datasets. Some datasets such as histone mass spectrometry or single-cell RNA-Seq require log-normalization of abundance/expression scores, and hence there is pre-processing of the data structure/matrix required. The number of parameters will also vary from one dataset to another depending on the variables of interest. While numerous network inference methods exist ranging from discrete-time networks (e.g., Boolean networks) to continuous-time/longitudinal network analysis, only graph network complexity is listed for illustration purposes. Further, the number of observations is subjected to debate as rich is a qualitative term with system-dependence (e.g., in single-cell datasets, rich implies hundreds if not thousands of cells per sample). Further, it is mentioned if the technique requires a priori assumptions such as fitting the dataset/observations to some statistical distribution.

SOURCE CODE FOR DETECTION TOOLS:

1) CODING AND BLOCK DECOMPOSITION METHOD:

The algorithmic complexity calculator: BDM can be computed on binarized and normalized adjacency matrices (e.g., gene expression matrices) or binary arrays:

http://complexitycalculator.com/

https://www.algorithmicdynamics.net/software.html

https://pybdm-docs.readthedocs.io/en/latest/

2) CONVERGENT CROSS MAPPING (TIME-DELAY EMBEDDING):

https://mran.microsoft.com/snapshot/2018-06-22/web/packages/rEDM/vignettes/rEDM-tutorial.html (Sugihara et al.) (rEDM package and tutorial for time-delay embedding)

3) LYAPUNOV EXPONENTS:

https://pypi.org/project/nolds/

https://www.mathworks.com/help/predmaint/ref/lyapunovexponent.html

https://blog.abhranil.net/2014/07/22/calculating-the-lyapunov-exponent-of-a-time-series-with-python-code/

4) **FRACTAL DIMENSION (BOX-COUNTING ALGORITHM):** (step by step guide and code for FD calculation)

Frederic Moisy (2021). boxcount (https://www.mathworks.com/matlabcentral/fileexchange/13063-boxcount), MATLAB Central File Exchange.

5) RC COMPUTING:

https://github.com/pvlachas/RNN-RC-Chaos

The public version in the above provided GitHub supports forecasting of multi-dimensional time-series.

6) FAST-FOURIER TRANSFORM:

https://towardsdatascience.com/fast-fourier-transform-937926e591cb

Bulti-in MATLAB FFT function, use fft (x), for some data matrix x.

7) MULTIFRACTAL ANALYSIS:

https://www.mathworks.com/matlabcentral/fileexchange/39069-hurst-exponent-estimation
